# Supplementary material for: Adolescent sexual health interventions that include very young adolescents in sub-Saharan Africa: a scoping review protocol
Source: BMJ Open. 2022 Nov 15;12(11):e063343. doi: 10.1136/bmjopen-2022-063343 (PMC9668002; doi:10.1136/bmjopen-2022-063343)
Supplement: Supplementary data [file bmjopen-2022-063343supp001.pdf]

Below is summary of initial searches done

Date	Search engine used	Number of publications retrieved
17/10/21	CINAHL(Ebsco)	1,927
17/09/21	Cochrane Library	1,007
17/09/21	Ovid MEDLINE	2,055
17/09/21	Ovid EMBASE	2,576
17/10/21	Global Index Medicus	1,752

Embase Classic+Embase <1947 to 2021 October 11>

1	exp adolescent behavior/ or exp adolescent mother/ or exp adolescent development/ or exp adolescent health/ or exp adolescent parent/ or exp adolescent sexual behavior/ or exp adolescent disease/ or exp adolescent pregnancy/ or exp adolescent/ or exp adolescent father/	1794522
2	exp sexual health/ or exp sexual behavior/ or exp sexuality/ or exp sexually transmitted disease/ or exp family planning/ or exp sexual education/	429495
3	exp pregnancy/ or exp reproductive health/	856202
4	exp child health care/	100563
5	exp Human immunodeficiency virus infection/	392472
6	exp adolescent pregnancy/	9963
7	(''sexual* health'' or sex* education or ''reproduct* health'' or pregnan* or contracept* or ''familly planning'' or HIV infections'' or ''Adolescen* behaviors'' or ''sexual behavior'' or ''child health care'' or ''adolescent development'' or ''adolescent behavior'' or ''adolescent mother'' or ''adolescent development'' or ''adolescent health'' or ''adolescent parent'' or ''adolescent disease'' or ''adolescent father'').tw,ti,ab.	887005
8	1 or 2 or 3 or 4 or 5 or 6 or 7	3588786

9	exp program development/ or exp program evaluation/	51829
10	exp health promotion/ or exp health education/	351668
11	(comprehensive or integrated or multilevel or multifaceted or multi component or multifaceted or multicomponent or multidimensional or multidimensional or holistic or ''holistic health'' or ''Community Networks'' or ''youth development program*'' or ''youth program*'' or ''teen program*'' or adolescent program* or ''opportunity programme'' or ''health promot*'' or ''program evaluation'').tw,ti,ab.	988657
12	9 or 10 or 11	1325483
13	8 and 12	172938
14	(teen* or youth* or adolescen* or juvenile* or (young adj2 (adult* or person* or individual* or people* or population* or man or men or wom*n)) or youngster* or first-grader* or second-grader* or third-grader* or fourth-grader* or fifth-grader* or sixth-grader* or seventh-grader* or highschool* or college* or ((secondary or high*) adj2 (school* or education))).tw,ti,ab.	1126352
15	13 and 14	49877
16	exp Africa, Central/ or exp Africa, Eastern/ or exp Africa, Western/ or exp Africa/ or exp "Africa South of the Sahara"/ or exp Africa, Southern/ or exp South Africa/	387913
17	15 and 16	2789
18	limit 17 to (human and yr="2000 -Current")	2576

Ovid MEDLINE: Epub Ahead of Print, In-Process & Other Non-Indexed Citations, Ovid MEDLINE® Daily and Ovid MEDLINE® <1946-Present>

1	exp Adolescent Behavior/ or exp Adolescent Health Services/ or exp Adolescent/ or exp Adolescent Health/ or exp Adolescent Development/	2127695
2	exp Sexual Health/ or exp Reproductive Health/ or exp Sexual Behavior/ or exp Sex Education/	122155
3	exp Reproductive Health/ or exp "Delivery of Health Care"/ or exp Family Planning Services/	1169678
4	exp Sexual Behavior/	113345

5	exp Pregnancy in Adolescence/	8244
6	exp HIV Infections/	296978
7	(''sexual* health'' or sex* education or ''reproduct* health'' or pregnan* or contracept* or ''familly planning'' or HIV infections'' or ''Adolescen* behaviors'' or ''sexual behavior'' or ''child health care'' or ''adolescent development'' or ''adolescent behavior'' or ''adolescent mother'' or ''adolescent development'' or ''adolescent health'' or ''adolescent parent'' or ''adolescent disease'' or ''adolescent father'').tw,ti,ab.	637818
8	1 or 2 or 3 or 4 or 5 or 6 or 7	3919310
9	exp program development/ or exp program evaluation/	100752
10	exp health promotion/ or exp health education/	254114
11	(comprehensive or integrated or multilevel or multifaceted or multi component or multifaceted or multicomponent or multidimensional or multidimensional or holistic or ''holistic health'' or ''Community Networks'' or ''youth development program*'' or ''youth program*'' or ''teen program*'' or adolescent program* or ''opportunity programme'' or ''health promot*'' or ''program evaluation'').tw,ti,ab.	779773
12	9 or 10 or 11	1072828
13	8 and 12	282250
14	(teen* or youth* or adolescen* or juvenile* or (young adj2 (adult* or person* or individual* or people* or population* or man or men or wom*n)) or youngster* or first-grader* or second-grader* or third-grader* or fourth-grader* or fifth-grader* or sixth-grader* or seventh-grader* or highschool* or college* or ((secondary or high*) adj2 (school* or education))).tw,ti,ab.	783907
15	13 and 14	48396
16	exp Africa, Central/ or exp Africa, Eastern/ or exp Africa, Western/ or exp Africa/ or exp "Africa South of the Sahara"/ or exp Africa, Southern/ or exp South Africa/	290672
17	15 and 16	2447
18	limit 17 to (humans and yr="2000 -Current")	2055

Cochrane

ID	Search	Hits
#1	MeSH descriptor: [Adolescent] explode all trees	107314
#2	MeSH descriptor: [Pregnancy in Adolescence] explode all trees	199
#3	MeSH descriptor: [Adolescent Behavior] explode all trees	1504
#4	MeSH descriptor: [Sexual Health] explode all trees	69
#5	MeSH descriptor: [Family Planning Services] explode all trees	256
#6	MeSH descriptor: [Reproductive Health] explode all trees	91
#7	MeSH descriptor: [HIV Infections] explode all trees	13092
#8	(''sexual* health'' or sex* education or ''reproduct* health'' or pregnan* or contracept* or ''familly planning'' or HIV infections'' or ''Adolescen* behaviors'' or ''sexual behavior'' or ''child health care'' or ''adolescent development'' or ''adolescent behavior'' or ''adolescent mother'' or ''adolescent development'' or ''adolescent health'' or ''adolescent parent'' or ''adolescent disease'' or ''adolescent father''):ti,ab,kw	164702
#9	#1 OR #2 OR #3 OR #4 OR #5 OR #6 OR #7 OR #8	226282
#10	MeSH descriptor: [Program Development] explode all trees	730
#11	MeSH descriptor: [Program Evaluation] explode all trees	6425
#12	(comprehensive or integrated or multilevel or multifaceted or multi component or multifaceted or multicomponent or multidimensional or multidimensional or holistic or ''holistic health'' or ''Community Networks'' or ''youth development program*'' or ''youth program*'' or ''teen program*'' or adolescent program* or ''opportunity programme'' or ''health promot*'' or ''program evaluation''):ti,ab,kw	97084
#13	#10 OR #11 OR #12	97312
#14	#9 AND #13	27099
#15	((teen* or youth* or adolescen* or juvenile* or (young adj2 (adult* or person* or individual* or people* or population* or man or men or wom*n)) or youngster* or first-grader* or second-grader* or third-grader* or fourth-grader* or fifth-grader* or sixth-grader* or seventh-grader* or highschool* or college* or ((secondary or high*) adj2 (school* or education)))):ti,ab,kw	166583

#16	#14 AND #15	18460
#17	(sub-Sahara* Africa OR West Africa OR East Africa OR Central Africa OR South* Africa OR SSA OR Cameroon OR "Central African Republic" OR Chad OR Congo OR "Democratic Republic of the Congo" OR Gabon OR Burundi OR Djibouti OR Eritrea OR Ethiopia OR Kenya OR Rwanda OR Somalia OR Sudan OR Tanzania OR Uganda OR Angola OR Botswana OR Lesotho OR Malawi OR Mozambique OR Namibia OR Swaziland OR Zambia OR Zimbabwe OR Benin OR Burkina Faso OR Cape Verde OR Cote d'Ivoire OR Gambia OR Ghana OR Guinea OR Equatorial Guinea OR Guinea-Bissau OR Liberia OR Mali OR Mauritania OR Niger OR Nigeria OR Senegal OR Sierra Leone OR Togo OR Comoros OR Madagascar OR Mauritius OR Seychelles OR Solomon Islands OR Marshall Islands OR Sao Tome and Principe):ti,ab,kw	20737
#18	#16 AND #17 with Publication Year from 2000 to 2021, with Cochrane Library publication date Between Jan 2000 and Dec 2021, in Trials (Word variations have been searched)	1007

Ebsco

Accessibility Information and Tips

Print Search History

MY EBSCOhost

Sunday, October 17, 2021 9:48:02 AM

#	Query	Limiters/Expanders	Last Run Via	Results
S23	S18 AND S19	Limiters - Published Date: 20000101-20211231<br>Expanders - Apply equivalent subjects<br>Search modes - Boolean/Phrase	Interface - EBSCOhost Research Databases<br>Search Screen - Advanced Search<br>Database - CINAHL Plus with Full Text	1,927

S22	S18 AND S19	Expanders - Apply equivalent subjects<br>Search modes - Boolean/Phrase	Interface - EBSCOhost Research Databases<br>Search Screen - Advanced Search<br>Database - CINAHL Plus with Full Text	1,952
S21	S18 AND S19	Limiters - Published Date: 20000101-20211231<br>Expanders - Apply equivalent subjects<br>Search modes - Boolean/Phrase	Interface - EBSCOhost Research Databases<br>Search Screen - Advanced Search<br>Database - CINAHL Plus with Full Text	1,927
S20	S18 AND S19	Expanders - Apply equivalent subjects<br>Search modes - Boolean/Phrase	Interface - EBSCOhost Research Databases<br>Search Screen - Advanced Search<br>Database - CINAHL Plus with Full Text	1,952
S19	(MH "South Africa") OR (MH "Namibia") OR (MH "Africa South of the Sahara")	Expanders - Apply equivalent subjects<br>Search modes - Boolean/Phrase	Interface - EBSCOhost Research Databases<br>Search Screen - Advanced Search<br>Database - CINAHL Plus with Full Text	24,636
S18	S16 AND S17	Expanders - Apply equivalent subjects<br>Search modes - Boolean/Phrase	Interface - EBSCOhost Research Databases<br>Search Screen - Advanced Search<br>Database - CINAHL Plus with Full Text	137,234
S17	TX (teen* OR youth* OR adolescen* OR juvenile* OR (young N2 (adult* OR person* OR individual* OR people* OR population* OR man OR men OR wom*n)) OR youngster* OR first-grader* OR second-grader* OR third-grader* OR fourth-grader* OR	Expanders - Apply equivalent subjects<br>Search modes - Boolean/Phrase	Interface - EBSCOhost Research Databases<br>Search Screen - Advanced Search<br>Database - CINAHL Plus with Full Text	1,853,657

	fifth-grader* OR sixth-grader* OR seventh-grader* OR highschool* OR college* OR ((secondary or high*) N2 (school* OR education)))			
S16	S11 AND S15	Expanders - Apply equivalent subjects Search modes - Boolean/Phrase	Interface - EBSCOhost Research Databases Search Screen - Advanced Search Database - CINAHL Plus with Full Text	171,808
S15	S12 OR S13 OR S14	Expanders - Apply equivalent subjects Search modes - Boolean/Phrase	Interface - EBSCOhost Research Databases Search Screen - Advanced Search Database - CINAHL Plus with Full Text	746,708
S14	TX comprehensive OR integrated OR multilevel OR multifaceted OR ''multi component'' OR multifaceted OR multicomponent OR multidimensional OR multidimensional'' OR holistic OR ''holistic health'' OR ''Community Networks'' OR ''youth development program*'' OR ''youth program*'' OR ''teen program*'' OR ''adolescent program*'' OR ''opportunity programme'' OR ''health promot*'' OR ''program evaluation''	Expanders - Apply equivalent subjects Search modes - Boolean/Phrase	Interface - EBSCOhost Research Databases Search Screen - Advanced Search Database - CINAHL Plus with Full Text	731,345
S13	(MH "Program Evaluation")	Expanders - Apply equivalent subjects Search modes - Boolean/Phrase	Interface - EBSCOhost Research Databases Search Screen - Advanced Search	44,498

			Database - CINAHL Plus with Full Text	
S12	(MH "Program Development")	Expanders - Apply equivalent subjects Search modes - Boolean/Phrase	Interface - EBSCOhost Research Databases Search Screen - Advanced Search Database - CINAHL Plus with Full Text	27,514
S11	S1 OR S2 OR S3 OR S4 OR S5 OR S6 OR S7 OR S8 OR S9 OR S10	Expanders - Apply equivalent subjects Search modes - Boolean/Phrase	Interface - EBSCOhost Research Databases Search Screen - Advanced Search Database - CINAHL Plus with Full Text	1,051,780
S10	TX ''sexual* health'' OR ''sex* education'' OR ''reproduct* health'' OR pregnan* OR contracept* OR ''familly planning'' OR ''HIV infections'' OR ''Adolescen* behaviors'' OR ''sexual behavior'' OR ''child health care'' OR ''adolescent development'' OR ''adolescent behavior'' OR ''adolescent mother'' OR ''adolescent development'' OR ''adolescent health'' OR ''adolescent parent'' OR ''adolescent disease'' OR ''adolescent father''	Expanders - Apply equivalent subjects Search modes - Boolean/Phrase	Interface - EBSCOhost Research Databases Search Screen - Advanced Search Database - CINAHL Plus with Full Text	596,080
S9	(MH "HIV Infections+") OR (MH "Human Immunodeficiency Virus+")	Expanders - Apply equivalent subjects Search modes - Boolean/Phrase	Interface - EBSCOhost Research Databases Search Screen - Advanced Search Database - CINAHL Plus with Full Text	92,927
S8	(MH "Family Planning") OR (MH "Family	Expanders - Apply equivalent subjects	Interface - EBSCOhost Research Databases	7,446

	Planning, Natural") OR (MH "Family Planning: Unplanned Pregnancy (Iowa NIC)") OR (MH "Family Planning: Contraception (Iowa NIC)") OR (MH "Family Planning (Omaha)")	Search modes - Boolean/Phrase	Search Screen - Advanced Search<br>Database - CINAHL Plus with Full Text	
S7	(MH "Sexual Behavior")	Expanders - Apply equivalent subjects<br>Search modes - Boolean/Phrase	Interface - EBSCOhost Research Databases<br>Search Screen - Advanced Search<br>Database - CINAHL Plus with Full Text	694
S6	(MH "Reproductive Health")	Expanders - Apply equivalent subjects<br>Search modes - Boolean/Phrase	Interface - EBSCOhost Research Databases<br>Search Screen - Advanced Search<br>Database - CINAHL Plus with Full Text	8,101
S5	(MH "Sex Education")	Expanders - Apply equivalent subjects<br>Search modes - Boolean/Phrase	Interface - EBSCOhost Research Databases<br>Search Screen - Advanced Search<br>Database - CINAHL Plus with Full Text	5,793
S4	(MH "Sexual Health") OR (MH "Sexually Transmitted Diseases, Viral")	Expanders - Apply equivalent subjects<br>Search modes - Boolean/Phrase	Interface - EBSCOhost Research Databases<br>Search Screen - Advanced Search<br>Database - CINAHL Plus with Full Text	7,940
S3	(MH "Sexually Transmitted Diseases, Viral")	Expanders - Apply equivalent subjects<br>Search modes - Boolean/Phrase	Interface - EBSCOhost Research Databases<br>Search Screen - Advanced Search<br>Database - CINAHL Plus with Full Text	393
S2	(MH "Adolescent Parents") OR (MH	Expanders - Apply equivalent subjects	Interface - EBSCOhost Research Databases	27,742

	"Adolescent Mothers") OR (MH "Adolescent Health Services") OR (MH "Adolescent Fathers") OR (MH "Adolescent Behavior") OR (MH "Adolescent Health")	Search modes - Boolean/Phrase	Search Screen - Advanced Search<br>Database - CINAHL Plus with Full Text	
S1	(MH "Adolescent Parents") OR (MH "Child Development: Adolescence (12-17 Years) (Iowa NOC)") OR (MH "Adolescence") OR (MH "Pregnancy in Adolescence") OR (MH "Maternal Age 14 and Under")	Expanders - Apply equivalent subjects<br>Search modes - Boolean/Phrase	Interface - EBSCOhost Research Databases<br>Search Screen - Advanced Search<br>Database - CINAHL Plus with Full Text	563,120

Global Index Medicus

search		
1	tw:((tw:(adolescent)) OR (tw:("family planning")) OR (tw:("sexual health")) OR (tw:(adolescent pregnancy")) OR (tw:("adolescent behavior")) OR (tw:(sexuality)) OR (tw:("sexual behavior")) OR (tw:("hiv infection"))) AND (collection_gim:("AIM")) AND (year_cluster:[2000 TO 2021])	1752
