# Supplementary material for: Adolescent sexual health interventions that include very young adolescents in sub-Saharan Africa: a scoping review protocol
Source: BMJ Open. 2022 Nov 15;12(11):e063343. doi: 10.1136/bmjopen-2022-063343 (PMC9668002; doi:10.1136/bmjopen-2022-063343)
Supplement: Author's manuscript [file bmjopen-2022-063343.draft_revisions.pdf]

BMJ Open is committed to open peer review. As part of this commitment we make the peer review history of every article we publish publicly available.

When an article is published we post the peer reviewers' comments and the authors' responses online. We also post the versions of the paper that were used during peer review. These are the versions that the peer review comments apply to.

The versions of the paper that follow are the versions that were submitted during the peer review process. They are not the versions of record or the final published versions. They should not be cited or distributed as the published version of this manuscript.

BMJ Open is an open access journal and the full, final, typeset and author-corrected version of record of the manuscript is available on our site with no access controls, subscription charges or pay-per-view fees (http://bmjopen.bmj.com).

If you have any questions on BMJ Open's open peer review process please email info.bmjopen@bmj.com

BMJ Open

Adolescent Sexual health interventions that include very young adolescents in sub-Saharan Africa: A scoping review protocol

Journal:	*BMJ Open*
Manuscript ID	bmjopen-2022-063343
Article Type:	Protocol
Date Submitted by the Author:	29-Mar-2022

Complete List of Authors:	Manda, Wanangwa; African Population and Health Research Center, CARTA; Kamuzu University of Health Sciences, Public Health<br>Sikweyiya, Yandisa; South African Medical Research Council, Gender & Health Research Unit; University of the Witwatersrand, School of Public Health<br>Kaunda-Khangamwa, Blessings; Kamuzu University of Health Sciences, Public Health<br>Selemani, Apatsa; Kamuzu University of Health Sciences, Public Health<br>Jimu, Scholastica; University of Benin, Obstetrics and Gynecology<br>Kamndaya, Mphatso; Malawi University of Business and Applied Sciences, Applied Sciences

Keywords:	Child & adolescent psychiatry < PSYCHIATRY, HIV & AIDS < INFECTIOUS DISEASES, PUBLIC HEALTH

SCHOLARONE™
Manuscripts

Adolescent Sexual health interventions that include very young adolescents in sub-Saharan Africa: A scoping review protocol

Wanangwa Chimwaza Manda[1,2], Yandisa Sikweyiya[3], Blessings Nyasilia Kaunda-Khangamwa[4], Apatsa Selemani[5], Scholastica Eunice Jimu[6], Mphatso Kamndaya[7]

[1] School of Public Health, University of the Witwatersrand, Johannesburg, South Africa, email:wmanda@cartafrica.org

[2] School of Public Health, Kamuzu University of Health Sciences, Blantyre, Malawi, email:wmanda@cartafrica.org

[3]Gender and Health Research Unit, South African Medical Research Council, Pretoria, South Africa, email: Yandisa.Sikweyiya@mrc.ac.za

[4] School of Public Health, Kamuzu University of Health Sciences, Blantyre, Malawi, email: b.n.kaunda@gmail.com

[5] School of Public Health, Kamuzu University of Health Sciences, Blantyre, Malawi, email: aselemani@cartafrica.org

[6] Department of Obstetrics and Gynecology, University of Benin, Benin, Nigeria, email: jimuschola@gmail.com

[7] School of Applied Sciences, Malawi University of Business and Applied Sciences, Blantyre, Malawi, email: mkamndaya@cartafrica.org

Corresponding Author*: Wanangwa Chimwaza Manda, Kamuzu University of Health Sciences, Private Bag 360, Chichiri, Blantyre 3. Telephone: 265999445465

For peer review only

For peer review only - http://bmjopen.bmj.com/site/about/guidelines.xhtml

Abstract

Introduction

Targeting very young adolescents (VYAs) with sexual health (SH) interventions is increasingly being recognized as one of the solutions to tackling SH challenges in late adolescence. However, there is a dearth of literature regarding SH interventions implemented specifically for VYAs in sub-Saharan Africa (SSA). This scoping review aims to give a summary of documented evidence on SH interventions that include VYAs in sub-Saharan Africa, identify gaps in existing interventions, and provide recommendations for further programmatic work on SH for VYAs.

Methods and analysis

The methods of this scoping review will be guided by the framework proposed by Arksey and O'Malley and further enhanced by Levac et al. and the Joanna Briggs Institute. We will search electronic databases: Popline, EMBASE, PubMed, CINAHL, Dimensions, African Journals Online (AJOL), and summon-country specific search. We will include only published studies from SSA and only adolescent SH interventions. The data will be charted using the Preferred Reporting Items for Systematic Review and Meta-Analysis Extension for Scoping Review (PRISMA-ScR). The data will then be collated and summarized.

Ethics and dissemination

The scoping review methodology involves putting together information from articles which are publicly available, this study does not require ethical approval. Findings of this scoping review will be published in a scientific journal and presented at relevant scientific fora and conferences. This scoping review will provide a comprehensive overview of the evidence base of adolescent SH interventions for VYAs in SSA and will highlight critical gaps in the existing interventions and areas where further programmatic work is needed for VYAs in SSA.

Registration: https://archive.org/details/osf-registrations-gn538-v1

Keywords: Adolescent, very young adolescents, sexual health, Interventions, sub-Saharan Africa

For peer review only - http://bmjopen.bmj.com/site/about/guidelines.xhtml

Article Summary

Strengths and limitation of the study

- This will be the first scoping review aiming to give a summary of documented evidence on SH interventions that include VYAs in sub-Saharan Africa
- The search strategy includes a range of electronic databases with peer-reviewed literature, including Popline, EMBASE, PubMed, CINAHL, Dimensions, African Journals Online (AJOL), Google. Directory of Open Access Journals (DOAJ) and summon-country specific search.
- Challenges are expected in finding published articles on VYAs SH interventions since research on VYAs has limited until recent years.
- Since we are focusing on interventions and programs of which the implementers are usually not researchers, it is highly likely that some interventions or programs involving VYAs may not have been published.
- Stakeholders will be consulted and engaged to provide insights on interventions which may not have been published or missed by the scoping review.

Background

The United Nations Children's Fund (UNICEF) defines adolescence as the period between the ages of 10 and 19 (1). UNICEF categorizes adolescents into very young adolescents (VYAs) aged 10 to 14 and older adolescents aged 15 to 19 (1). There are about 1.2 billion adolescents globally, half of which are VYAs (2). About 90% of these adolescents live in low and middle-income countries (LMICs) (3). The period of adolescence is considered a time of transition from childhood to adulthood which encompasses rapid physical, biological and intellectual changes (4–6). The period of adolescence is also considered as the period during which the individual progresses from the initial appearance of secondary sex characteristics e.g. increased facial, chest or pubic hair and upper body muscle for boys, while girls show rounded figure/hips, breasts budding, and the onset of the menstrual cycle to that of sexual maturity (7,8). During this period psychological processes and patterns of identification develop from those of a child to an independent unique adult (9). As the biological, cognitive, psychosocial, and emotional changes are happening the adolescents also

For peer review only - http://bmjopen.bmj.com/site/about/guidelines.xhtml

experience changes in comprehending life, their relationships, spaces and supports they interact with across adolescence (10).

Globally, adolescents face a myriad of challenges such as onset of sexual attention, sexual thoughts and experimentation, sexual violence, sexual coercion and sexual abuse (11–17). Most of these challenges are related to their sexual health (SH) most of which arise because of sexual risk behaviours in adolescence (11–17). Some of the risky behaviours include premarital sex, early sexual initiation, unprotected sexual intercourse, sex with multiple partners (11–16). These risky behaviours result in negative sexual and reproductive health outcomes such as early and unwanted pregnancies, unsafe abortion and sexually transmitted infections including HIV. Every year, an estimated 21 million girls aged 15 to 19 years and 2 million VYA girls become pregnant in developing regions (18). Every year, some 3.9 million girls aged 15 to 19 years undergo unsafe abortions globally (19). It is estimated that in 2016, there were almost 2.1 million adolescents living with HIV including 770000 VYA and 1.3 million older adolescents globally (20).

Even though the majority of VYA are not yet engaging in penetrative sex, their sexual curiosities have begun and some are reported to indulge in non-penetrative sexual activities such kissing, fondling, foreplay, and heavy petting (2,21–23). In addition a few studies in SSA that have been conducted among young adolescents reveal that substantial numbers of boys and girls engage in unprotected heterosexual vaginal intercourse either by choice or coercion—before their 15th birthdays (24–27). These findings suggest that VYAs are at risk of negative SH outcomes. In addition, studies have shown that it is during early adolescence that adolescents begin to form new gender roles and sexual identities within their social contexts(28). As these developments occur, VYAs also acquire information, develop attitudes and experiment with behaviours and relationships (29). These developments and experiences might predispose or protect them from SH challenges such as early unintended pregnancies, STI infection including HIV (30).

In LMICs where most of these SH challenges occur, investment in positive youth development to promote sexual and reproductive health is increasing (22,31–34). However, most of the adolescent SH interventions focus on the older adolescents overlooking the SH needs of VYAs (2,22,31,35).

For peer review only - http://bmjopen.bmj.com/site/about/guidelines.xhtml

Not only are VYAs overlooked in SH interventions, gaps exist in information about SH interventions that target VYAs in sub-Saharan Africa (36,37).

Targeting VYAs with SH interventions is increasingly being recognized as one of the solutions to tackling SH challenges in late adolescence (28,31,38). As such, adolescent sexual and reproductive health programs are being implemented in various countries around the world through various platforms including family life education, youth centers, youth-friendly health services, peer education programs (29,31,37). However, there is a dearth of literature regarding sexual and reproductive health programs that include VYAs in sub-Saharan Africa (SSA). This scoping review aims to give a summary of documented evidence on SH interventions that include VYAs in sub-Saharan Africa, identify gaps in existing interventions, and provide recommendations for further programmatic work on SH for very young adolescents in SSA. It is envisaged that findings from this scoping review will contribute to the evidence that is required to develop evidence-based, culturally, and age-appropriate primary prevention programs and policies for addressing SH needs of VYA in SSA.

Specific objectives

1. To describe the types of SH interventions that have been implemented for VYAs in SSA
2. To describe the settings in which the SH interventions for VYAs have been implemented in SSA
3. To document the barriers and facilitators of implementing SH interventions for VYAs in SSA
4. To identify gaps in SH interventions implemented for VYAs in SSA
5. To provide recommendations for further programmatic work on SH for very young adolescents in SSA

Protocol design

The methods of this scoping review will be guided by the framework proposed by Arksey and O'Malley and further enhanced by Levac et al. and the Joanna Briggs Institute (39,40). The stages in the framework include:

- Stage 1. Identifying the research question.
- Stage 2. Identifying relevant studies.

For peer review only - http://bmjopen.bmj.com/site/about/guidelines.xhtml

- Stage 3. Study selection.
- Stage 4. Charting the data.
- Stage 5. Collating, summarising, and reporting the result
- Stage 6. Consultative forum

Stage 1: Identifying the research questions

This scoping review has the following research questions:

Main question

What evidence is there for SH interventions that have been implemented for VYAs in SSA?

Specific questions

1. What types of SH interventions have been implemented for VYAs in SSA?
2. In what settings have these interventions been implemented?
3. What have been the implementation barriers and facilitators of these SH interventions for VYAs
4. What are the gaps identified in these interventions?
5. What future programmatic work can be recommended on SH for very young adolescents in SSA?

Stage 2: Identifying relevant studies

To identify relevant studies for this review, a search for relevant articles and publications on adolescent sexual and reproductive health interventions in sub-Saharan Africa will be conducted using several electronic databases including the Popline, EMBASE, PubMed, CINAHL, Dimensions, African Journals Online (AJOL) and Summon-country specific search. Additional searches of key terms in Google Scholar and Directory of Open Access Journals (DOAJ) will be performed to find additional literature.

Several search techniques will be employed including phrase searching, Boolean operators (AND, OR, NOT), Truncation, Wildcats, Field search, Proximity search, citation chaining and filters to

For peer review only - http://bmjopen.bmj.com/site/about/guidelines.xhtml

limit searches to inclusion criteria presented in this protocol. The final search strings will be presented in the report. The search terms will include a combination of keywords and alternative terms such as: "Adolescent" OR "Very young Adolescents" OR "VYAs" OR "early adolescents" OR "Teenagers" OR "boys" OR "girls", AND "Sexual and Reproductive health" OR "Reproductive health" OR "sexual health" OR "reproductive health care" OR "contraception" OR teen pregnancy" OR "sex education", AND "Interventions" OR "Programming", AND "Sub-Saharan Africa" OR "Country name of a Sub-Saharan country". All researchers involved in the review will keep an updated record of the number of publications identified and date during each session of the literature search using table 1 below:

Table 1:Electronic search record

Date	Keyword searched	Search engine used	Number of publications retrieved

An academic librarian will be consulted to assist with identifying appropriate subject headings terms for the search and how to modify them for different databases that will be used. Relevant articles will be downloaded from the databases and uploaded into Mendeley (for importation and duplicates screening) first, then exported to Rayyan systematic review software for further screening of titles and abstracts.

3. Study Selection

According to the Arksey and O'Malley framework, the third stage of the process of the scoping review is to select studies to be included in the review (see figure 1 in the additional files section after the references). The first step in this third stage will involve the screening of the titles and abstracts of the peer-reviewed articles and publications that were downloaded in the second stage. The articles and publications will be screened according to the criteria that were used in the second stage which will include only studies from sub-Saharan Africa; only adolescent sexual and reproductive health interventions and all interventions that include adolescents. The second step in this process will be a review of full-text articles and publications whose titles and abstracts have been deemed relevant for this review and the screening will be done by two reviewers. Should

For peer review only - http://bmjopen.bmj.com/site/about/guidelines.xhtml
For peer review only

there be a disagreement between the two reviewers, the reviewers will consult with a third reviewer who will decide on whether to include the article or not.

Inclusion criteria

The focus of the review will be peer reviewed articles and publications from the sub-Saharan Africa from 2000-2022. Other criteria for inclusion will include language (only English articles and publications), type of Intervention (only sexual health and reproductive interventions) and study population (adolescents age 10-19). These criteria were informed by the Population–Concept–Context (PCC) framework (See table 2) recommended by the Joanna Briggs Institute for scoping reviews to determine the eligibility of research and is recommended as less restrictive alternative to the PICO (Population, Intervention, Comparator and Outcome) mnemonic recommended for systematic reviews.

Exclusion Criteria

The peer reviewed articles and publications from outside the sub-Saharan Africa, publications not in English, including publications before 2000 and after 2022 will not be included in this review.

Table 2: Eligibility of the research question

PCC Element	Definition
Population	The population for this study will be all adolescents from ages 10 to 19,
Concept	All sexual health interventions targeting adolescents published between 2000 and 2022
Context	Research articles and publications are limited to sub-Saharan Africa

4. Charting the data

The fourth stage of the process of this scoping review will involve charting of the data using Preferred Reporting Items for Systematic Review and Meta-Analysis Extension for Scoping review(PRISMA-ScR). This will involve extraction of study characteristics and putting this information in a table or a chart. The study characteristics that will be extracted will include: Author, title, year of publication, study location, type of intervention, duration of intervention,

For peer review only - http://bmjopen.bmj.com/site/about/guidelines.xhtml

study population, and key findings (see table 3). The chart developed will be piloted by two reviewers separately to make sure that the chart is capturing information consistently and accurately. The whole process of extracting data from the studies will be done independently by two reviewers. The charted data from the two reviewers will then be compared and any discrepancies will be further discussed to ensure consistency between the reviewers.

Table 3: Sample data extraction table

1. Authors
2. Title
3. Year of publication
4. Country of Origin
5. Aims of the study
6. Sample size
7. Methodology
8. Intervention
9. Outcomes measured
10. Duration of the study
11. Key findings which match the review questions

For peer review only

For peer review only - http://bmjopen.bmj.com/site/about/guidelines.xhtml

5. Collating, summarizing and reporting the result

This stage will involve collating, summarizing, and reporting the results from the process in Stage 4. This will provide summary information on what types of adolescent sexual and reproductive health interventions have been implemented for VYAs in sub-Saharan Africa, settings, effectiveness and implementation challenges and facilitators. In addition, this process will also show interventions that have been under researched and may require further investigation.

Stage 6: Consultation exercise

At this stage we will involve relevant stakeholders working in the field of Adolescent SH in Malawi. The stakeholders will be engaged to provide valuable insight beyond our scoping review report. Since these stakeholders will be experts working in the field of adolescent SH we envisage that that they will have the potential to add value to our work such as providing additional references and new insights to our findings. Therefore, we will organize an in-person workshop where we will present our findings to stakeholders and allow them to contribute their insights to our work.

Patient and public involvement

The design of this scoping review protocol did not involve patients or the public, but we will aim to involve relevant stakeholders working in the field of Adolescent SH in Malawi in the review process, consulting, discussing and verifying the results of this review.

Ethics and Dissemination

Since the scoping review methodology involves putting together information from articles which are publicly available, this study does not require ethical approval. To disseminate our findings, we will submit a manuscript reporting our findings for publication to a scientific journal. Furthermore, the findings of this scoping review will be presented in various scientific fora and conferences. It is anticipated that, the scoping review will provide a comprehensive overview of the evidence base of adolescent sexual health interventions for VYAs and will highlight areas where evidence is inconclusive or missing. The review will also provide key information to policy makers and health professionals interested in planning, funding, and delivering evidence based and effective sexual health interventions for VYAs.

For peer review only - http://bmjopen.bmj.com/site/about/guidelines.xhtml

Discussion

The proposed scoping review will generate a synthesis of sexual and reproductive health programs or interventions implemented that included VYAs in Sub-Saharan Africa. For a long time, SH programs have focused on older adolescents aged 15 to 19 years overlooking VYAs. (refs) As such, literature is replete with programs or interventions for older adolescents (28) As investments in positive youth development to promote SH is increasing (31), attention is shifting to VYAs such that there has been an increase in both VYA SH research and programming over the past five years, globally (2,24,27,35,41,42). However, there are very few VYAs SH interventions that have been documented especially in Sub-Saharan Africa (2,31).

Findings from this scoping review will be relevant for both policymakers and SH intervention programmers for VYAs in SSA. For instance, the synthesis of SH interventions that will be documented will showcase what SH interventions have been implemented for VYAs so far in SSA including the areas of focus, what works and what does not work in implementing these interventions. This knowledge is critical for developing evidence-based context-specific and culturally appropriate interventions for VYAs.

Furthermore, findings from this scoping review will be important for researchers working in the area of VYA SH as this scoping review has the potential of generating questions for further research around why some interventions work well and why others do not work. In addition, the scoping review has the potential of identifying gaps in SH interventions implemented for VYAs in SSA that may need further research.

Definition of key terms

Adolescence: The period between age 10 and 19

Adolescents: Young people from age 10 to 19

Very young adolescent: Adolescents ages 10 to 14 years

For peer review only - http://bmjopen.bmj.com/site/about/guidelines.xhtml

Older adolescents: Adolescents ages 15 to 19 years

Sexual health: a state of physical, emotional, mental, and social well-being in relation to sexuality; it is not merely the absence of disease, dysfunction or infirmity.

Authors Contributions

WCM conceived of the idea, developed the research questions and study methods and contributed meaningfully to the drafting and editing. BK, AS, and SJ helped in developing the research questions and study methods, contributed meaningfully to the drafting and editing. YS and MK supervised the preparation of the protocol and critically reviewed the manuscript for important intellectual content. All authors approved the final manuscript.

Funding

This study will be supported by the Consortium for Advanced Research Training in Africa (CARTA). CARTA is jointly led by the African Population and Health Research Center and the University of the Witwatersrand and funded by the Carnegie Corporation of New York (Grant No—G-19-57145), Sida (Grant No:54100113), Uppsala Monitoring Centre and the DELTAS Africa Initiative (Grant No: 107768/Z/15/Z). The DELTAS Africa Initiative is an independent funding scheme of the African Academy of Sciences (AAS)'s Alliance for Accelerating Excellence in Science in Africa (AESA) and supported by the New Partnership for Africa's Development Planning and Coordinating Agency (NEPAD Agency) with funding from the Wellcome Trust (UK) and the UK government. The statements made and views expressed are solely the responsibility of the Fellow.

Competing Interests

The authors declare that there are no competing interests

Availability of data and materials

All data will be published in a data repository and can be accessed from the repository.

Word Count:

For peer review only - http://bmjopen.bmj.com/site/about/guidelines.xhtml

References

1. Unicef. Adolescence An Age of Opportunity [Internet]. Unicef. 2011. 137 p. Available from: www.unicef.org

2. Woog V, Kågesten A. The Sexual and Reproductive Health Needs of Very Young Adolescents Aged 10–14 in Developing Countries: What Does the Evidence Show? 2017;(May). Available from: https://www.guttmacher.org/sites/default/files/report_pdf/srh-needs-very-young-adolescents-report_0.pdf

3. Nagata JM, Ferguson BJ, Ross DA. Research Priorities for Eight Areas of Adolescent Health in Low- and Middle-Income Countries. J Adolesc Heal. 2016;59(1).

4. Dixon-Mueller R. How young is "too young"? Comparative perspectives on adolescent sexual, marital, and reproductive transitions. In: Studies in Family Planning. 2008. p. 247–62.

5. Morris JL, Rushwan H. Adolescent sexual and reproductive health: The global challenges. Int J Gynecol Obstet [Internet]. 2015;131:S40–52. Available from: http://dx.doi.org/10.1016/j.ijgo.2015.02.006

6. Lerner RM, Galambos NL. ADOLESCENT DEVELOPMENT : Challenges and Opportunities for Research , Programs , and Policies. 1998;

7. Simpson a R. Raising Teens: A Synthesis of Research and a Foundation for Action. 2001;1–101. Available from: http://hrweb.mit.edu/worklife/rp_o

8. Kabiru CW, Beguy D, Undie C-C, Zulu EM, Ezeh AC. Transition into first sex among adolescents in slum and non-slum communities in Nairobi, Kenya. J Youth Stud [Internet]. 2010;13(4):453–71. Available from: http://www.tandfonline.com/doi/full/10.1080/13676261003801754

9. Counterparts G. Adolescent Health and Development (AHD) A Resource Guide for

For peer review only - http://bmjopen.bmj.com/site/about/guidelines.xhtml

World Bank Operations Staff. 2004.

10. Stang J, Story M. Chapter 1 ADOLESCENT GROWTH AND DEVELOPMENT. 2005;1–8.

11. Daliana N, Farid N, Rus SC, Dahlui M, Al-sadat N, Aziz NA. Predictors of sexual risk behaviour among adolescents from welfare institutions in Malaysia : a cross sectional study. BMC Public Health [Internet]. 2014;14(Suppl 3):S9. Available from: http://www.biomedcentral.com/1471-2458/14/S3/S9

12. Das AK. Reproductive Health of Indian Adolescents and Role of Educational Institutions : A Study Knowledge of Indian Adolescent about Sexual Health : Indian Institute of Social Reform & Research. 2015.

13. Albert, B., Brown, S., & Flanigan C. 14 and Younger: The Sexual Behavior of Young Adolescents (Summary). Washington, DC: National Campaign to Prevent Teen Pregnancy; 2003. p. 1.

14. Tangmunkongvorakul A, Carmichael G, Banwell C, Utomo ID, Sleigh A. Sexual perceptions and practices of young people in Northern Thailand. J Youth Stud [Internet]. 2011;14(3):315. Available from: http://www.ncbi.nlm.nih.gov/pubmed/22319025

15. Upadhyay UD, Hindin MJ. Do Perceptions of Friends' Behaviors Affect Age at First Sex? Evidence from Cebu, Philippines. J Adolesc Heal. 2006;39(4):570–7.

16. Clark S. Early Marriage and HIV Risks in Sub-Saharan Africa. Stud Fam Plann [Internet]. 2004;35(3):149–60. Available from: http://doi.wiley.com/10.1111/j.1728-4465.2004.00019.x

17. Mehta SD, Seeley J. Grand Challenges in Adolescent Sexual and Reproductive Health. Front Reprod Heal. 2020;2(June):2–5.

18. UNFPA. Girlhood, not motherhood: Preventing adolescent pregnancy [Internet]. New York: UNFPA; 2015. Available from: www.unfpa.org

19. Franjić S. Adolescent Pregnancy is a Serious Social Problem. J Gynecol Res Obstet.

For peer review only - http://bmjopen.bmj.com/site/about/guidelines.xhtml

2018;4(1):5–8.

20. UNFPA. Adolescent sexual and reproductive health. United Nations Popul Fund [Internet]. 2014;(August):1–18. Available from: http://www.unfpa.org/resources/adolescent-sexual-and-reproductive-health

21. Dixon-Mueller R. Starting young: Sexual initiation and HIV prevention in early adolescence. Vol. 13, AIDS and Behavior. 2009. p. 100–9.

22. Brady M. Calling attention to young adolescents: Building the evidence base to inform policies and programs. Transitions to Adulthood. 2011;(37):1–4.

23. Maina BW, Orindi BO, Sikweyiya Y, Kabiru CW. Gender norms about romantic relationships and sexual experiences among very young male adolescents in Korogocho slum in Kenya. Int J Public Health [Internet]. 2020;65(4):497–506. Available from: https://doi.org/10.1007/s00038-020-01364-9

24. Bankole A, Biddlecom A, Guiella G, Singh S, Zulu E. Sexual behavior, knowledge and information sources of very young adolescents in four sub-Saharan African countries. Afr J Reprod Health [Internet]. 2007;11(3):28–43. Available from: http://www.ncbi.nlm.nih.gov/pubmed/18458739%5Cnhttp://www.pubmedcentral.nih.gov/articlerender.fcgi?artid=PMC2367131

25. Atuyambe LM, Kibira SPS, Bukenya J, Muhumuza C, Apolot RR, Mulogo E. Understanding sexual and reproductive health needs of adolescents: evidence from a formative evaluation in Wakiso district, Uganda. Reprod Health [Internet]. 2015;12:35. Available from: http://www.ncbi.nlm.nih.gov/pubmed/25896066%5Cnhttp://www.pubmedcentral.nih.gov/articlerender.fcgi?artid=PMC4416389

26. Seifu A, Fantahun M, Worku A. Reproductive health needs of out-of-school adolescents : A cross-sectional comparative study of rural and urban areas in northwest Ethiopia. EthiopJHealth Dev. 2006;20(1):17.

27. Ortiz-Echevarria L, Greeley M, Bawoke T, Zimmerman L, Robinson C, Schlecht J.

For peer review only

For peer review only - http://bmjopen.bmj.com/site/about/guidelines.xhtml

Understanding the unique experiences, perspectives and sexual and reproductive health needs of very young adolescents: Somali refugees in Ethiopia. Confl Health. 2017;11(Suppl 1).

28. Maina BW, Izugbara C, Sikweyiya Y, Nandongwa I, Kabiru CW. Gender Norms and Sexual Behavior among Very Young Adolescents in Sub-Saharan Africa. Routledge Handb African Demogr. 2021;330–48.

29. Institute for Reproducive Health. Reaching very young adolescents (VYAs): Advancing program , research and evaluation practices [Internet]. Washington, D.C.; 2010. Available from: http://irh.org/wp-content/uploads/2013/04/IRH_VYA_Technical_Consultation_2010_Meeting_Report_FINAL.pdf

30. Save the Children. Building Consensus to Address Very Young Adolescents ' Sexual and Reproductive Health : A Guide to Planning and Hosting a Technical Consultation. 2015.

31. Igras SM, Macieira M, Murphy E, Lundgren R. Investing in very young adolescents' sexual and reproductive health. Glob Public Health [Internet]. 2014;9(5):555–69. Available from: http://www.ncbi.nlm.nih.gov/pubmed/24824757%5Cnhttp://www.pubmedcentral.nih.gov/articlerender.fcgi?artid=PMC4066908

32. Blum et al. A conceptual framework for early adolescence: a platform for research. Int J Adolesc Med Heal. 2015;33(4):395–401.

33. Sommer M. An overlooked priority: Puberty in sub-Saharan Africa. Am J Public Health. 2011;101(6):979–81.

34. Bank W. Adolescent health in the 21st century. 2011;14–5.

35. McCarthy K, Brady M, Hallman K. Investing When It Counts: Reviewing the Evidence and Charting a Course of Research and Action for Very Young Adolescents. 2016;1–69.

36. Lee C, Aules Y, Sami S, Lar PK, Schlect J, Robinson C. Sexual and reproductive health needs and risks of very young adolescent refugees and migrants from Myanmar living in

For peer review only - http://bmjopen.bmj.com/site/about/guidelines.xhtml

Thailand. Confl Health. 2017;11(Suppl 1).

37. WHO. Meeting report The sexual and reproductive health of young adolescents in developing countries : Who. 2010;

38. Odimegwu CO, Adewoyin Y. The Routledge Handbook of African Demography [Internet]. Katz WF, Assmann PF, editors. The Routledge Handbook of Demography. London: Routledge; 2021. 1–648 p. Available from: https://www.taylorfrancis.com/books/9780429508561

39. Arksey H OL. Scopins studies: towards a methodological framework . Int J Soc Res Methodol. 2005;8:19–32.

40. Fallis A. Reviewer's Manual. J Chem Inf Model [Internet]. 2013;53(9):1689–99. Available from: www.joannabriggs.org

41. Mmari K, A M, Blum RW, Ph D, H MP, Atnafou R, et al. Exploration of Gender Norms and Socialization Among Early Adolescents : The Use of Qualitative Methods for the Global Early Adolescent Study. J Adolesc Heal [Internet]. 2017;61(4):S12–8. Available from: http://dx.doi.org/10.1016/j.jadohealth.2017.07.006

42. Essop R, Tolla T, Lynch I, Makoae M. 'They tell you about the risks': Exploring sources of sexuality education among very young adolescents in rural Mpumalanga. SAJCH South African J Child Heal. 2018;12(Special Issue):S36–9.

For peer review only

For peer review only - http://bmjopen.bmj.com/site/about/guidelines.xhtml

For peer review only

For peer review only - http://bmjopen.bmj.com/site/about/guidelines.xhtml

Additional File : Study selection process

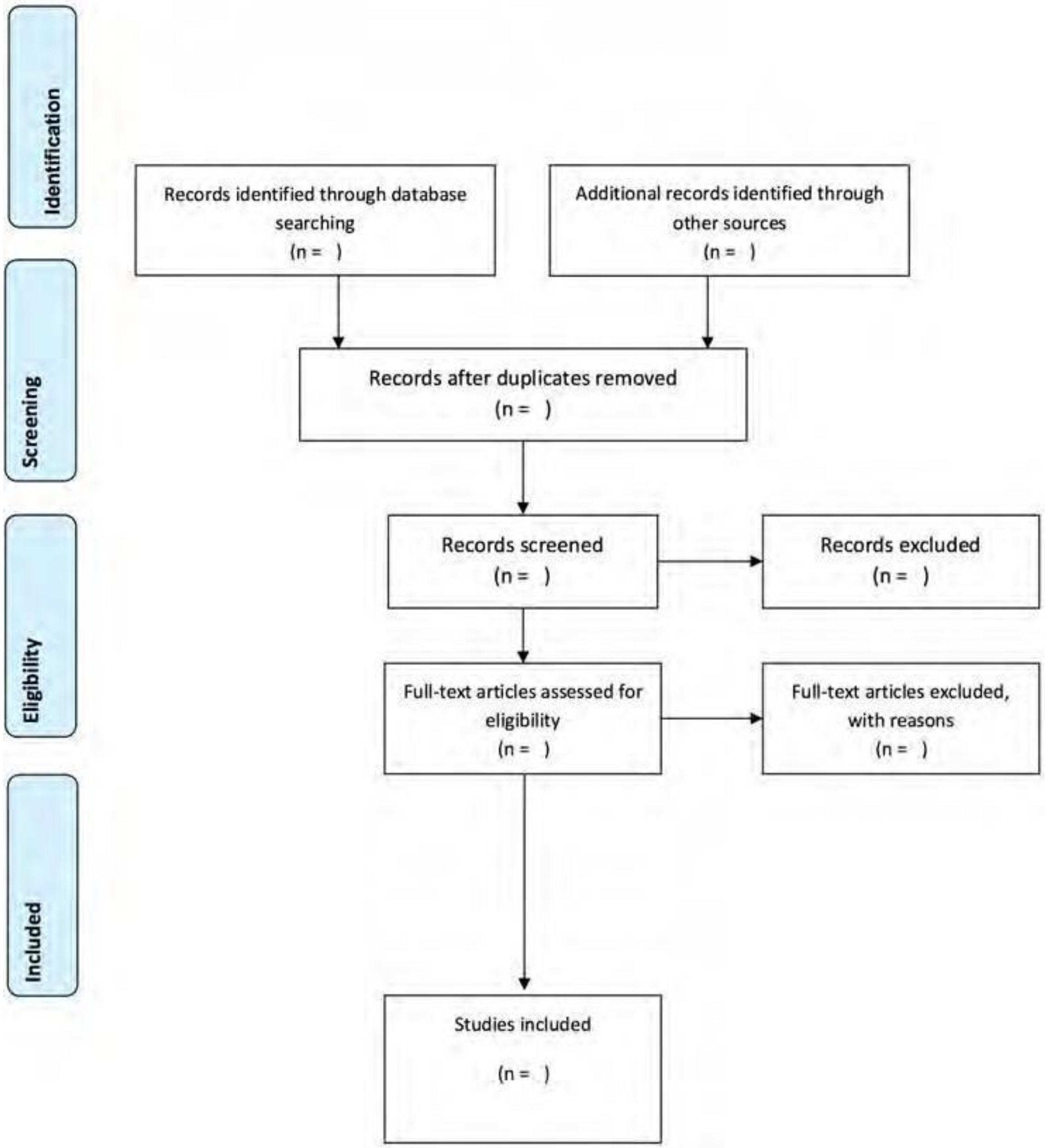

Figure 1. Flow diagram describing process of articles and publications being reviewed and selected

Preferred Reporting Items for Systematic reviews and Meta-Analyses extension for Scoping Reviews (PRISMA-ScR) Checklist

SECTION	ITEM	PRISMA-ScR CHECKLIST ITEM	REPORTED ON PAGE #
**TITLE**			
Title	1	Identify the report as a scoping review.	1
**ABSTRACT**			
Structured summary	2	Provide a structured summary that includes (as applicable): background, objectives, eligibility criteria, sources of evidence, charting methods, results, and conclusions that relate to the review questions and objectives.	2
**INTRODUCTION**			
Rationale	3	Describe the rationale for the review in the context of what is already known. Explain why the review questions/objectives lend themselves to a scoping review approach.	4
Objectives	4	Provide an explicit statement of the questions and objectives being addressed with reference to their key elements (e.g., population or participants, concepts, and context) or other relevant key elements used to conceptualize the review questions and/or objectives.	5-6
**METHODS**			
Protocol and registration	5	Indicate whether a review protocol exists; state if and where it can be accessed (e.g., a Web address); and if available, provide registration information, including the registration number.	2
Eligibility criteria	6	Specify characteristics of the sources of evidence used as eligibility criteria (e.g., years considered, language, and publication status), and provide a rationale.	8
Information sources*	7	Describe all information sources in the search (e.g., databases with dates of coverage and contact with authors to identify additional sources), as well as the date the most recent search was executed.	6-7
Search	8	Present the full electronic search strategy for at least 1 database, including any limits used, such that it could be repeated.	7
Selection of sources of evidence†	9	State the process for selecting sources of evidence (i.e., screening and eligibility) included in the scoping review.	7-8
Data charting process‡	10	Describe the methods of charting data from the included sources of evidence (e.g., calibrated forms or forms that have been tested by the team before their use, and whether data charting was done independently or in duplicate) and any processes for obtaining and confirming data from investigators.	8-9
Data items	11	List and define all variables for which data were sought and any assumptions and simplifications made.	9
Critical appraisal of individual sources of evidence§	12	If done, provide a rationale for conducting a critical appraisal of included sources of evidence; describe the methods used and how this information was used in any data synthesis (if appropriate).	Not done yet

For peer review only - http://bmjopen.bmj.com/site/about/guidelines.xhtml

SECTION	ITEM	PRISMA-ScR CHECKLIST ITEM	REPORTED ON PAGE #
Synthesis of results	13	Describe the methods of handling and summarizing the data that were charted.	10
**RESULTS**			
Selection of sources of evidence	14	Give numbers of sources of evidence screened, assessed for eligibility, and included in the review, with reasons for exclusions at each stage, ideally using a flow diagram.	7, 18
Characteristics of sources of evidence	15	For each source of evidence, present characteristics for which data were charted and provide the citations.	Not yet done
Critical appraisal within sources of evidence	16	If done, present data on critical appraisal of included sources of evidence (see item 12).	Not yet done
Results of individual sources of evidence	17	For each included source of evidence, present the relevant data that were charted that relate to the review questions and objectives.	Not yet done
Synthesis of results	18	Summarize and/or present the charting results as they relate to the review questions and objectives.	Not yet done
**DISCUSSION**			
Summary of evidence	19	Summarize the main results (including an overview of concepts, themes, and types of evidence available), link to the review questions and objectives, and consider the relevance to key groups.	Not yet done
Limitations	20	Discuss the limitations of the scoping review process.	3
Conclusions	21	Provide a general interpretation of the results with respect to the review questions and objectives, as well as potential implications and/or next steps.	Not yet done
**FUNDING**			
Funding	22	Describe sources of funding for the included sources of evidence, as well as sources of funding for the scoping review. Describe the role of the funders of the scoping review.	16

JBI = Joanna Briggs Institute; PRISMA-ScR = Preferred Reporting Items for Systematic reviews and Meta-Analyses extension for Scoping Reviews.

* Where *sources of evidence* (see second footnote) are compiled from, such as bibliographic databases, social media platforms, and Web sites.

† A more inclusive/heterogeneous term used to account for the different types of evidence or data sources (e.g., quantitative and/or qualitative research, expert opinion, and policy documents) that may be eligible in a scoping review as opposed to only studies. This is not to be confused with *information sources* (see first footnote).

‡ The frameworks by Arksey and O'Malley (6) and Levac and colleagues (7) and the JBI guidance (4, 5) refer to the process of data extraction in a scoping review as data charting.

§ The process of systematically examining research evidence to assess its validity, results, and relevance before using it to inform a decision. This term is used for items 12 and 19 instead of "risk of bias" (which is more applicable to systematic reviews of interventions) to include and acknowledge the various sources of evidence that may be used in a scoping review (e.g., quantitative and/or qualitative research, expert opinion, and policy document).

From: Tricco AC, Lillie E, Zarin W, O'Brien KK, Colquhoun H, Levac D, et al. PRISMA Extension for Scoping Reviews (PRISMAScR): Checklist and Explanation. Ann Intern Med. 2018;169:467–473. doi: 10.7326/M18-0850.

For peer review only - http://bmjopen.bmj.com/site/about/guidelines.xhtml

BMJ Open

Adolescent Sexual health interventions that include very young adolescents in sub-Saharan Africa: A scoping review protocol

Journal:	*BMJ Open*
Manuscript ID	bmjopen-2022-063343.R1
Article Type:	Protocol
Date Submitted by the Author:	31-Aug-2022
Complete List of Authors:	Manda, Wanangwa; African Population and Health Research Center, CARTA; Kamuzu University of Health Sciences, Public Health<br>Sikweyiya, Yandisa; South African Medical Research Council, Gender & Health Research Unit; University of the Witwatersrand, School of Public Health<br>Kaunda-Khangamwa, Blessings; Kamuzu University of Health Sciences, Public Health<br>Selemani, Apatsa; University of the Witwatersrand Johannesburg, Public Health<br>Jimu, Scholastica; University of Benin, Obstetrics and Gynecology<br>Kamndaya, Mphatso; Malawi University of Business and Applied Sciences, Applied Sciences
**Primary Subject Heading**:	Sexual health
Secondary Subject Heading:	Public health
Keywords:	Child & adolescent psychiatry < PSYCHIATRY, HIV & AIDS < INFECTIOUS DISEASES, PUBLIC HEALTH

SCHOLARONE™
Manuscripts

Adolescent Sexual health interventions that include very young adolescents in sub-Saharan Africa: A scoping review protocol

Wanangwa Chimwaza Manda[1,2], Yandisa Sikweyiya[3], Blessings Nyasilia Kaunda-Khangamwa[4], Apatsa Selemani[5], Scholastica Eunice Jimu[6], Mphatso Kamndaya[7]

[1] School of Public Health, University of the Witwatersrand, Johannesburg, South Africa, email:wmanda@cartafrica.org

[2] School of Public Health, Kamuzu University of Health Sciences, Blantyre, Malawi, email:wmanda@cartafrica.org

[3]Gender and Health Research Unit, South African Medical Research Council, Pretoria, South Africa, email: Yandisa.Sikweyiya@mrc.ac.za

[4] School of Public Health, Kamuzu University of Health Sciences, Blantyre, Malawi, email: b.n.kaunda@gmail.com

[5] School of Public Health, University of the Witwatersrand, Johannesburg, South Africa, email: aselemani@cartafrica.org

[6] Department of Obstetrics and Gynecology, University of Benin, Benin, Nigeria, email: jimuschola@gmail.com

[7] School of Applied Sciences, Malawi University of Business and Applied Sciences, Blantyre, Malawi, email: mkamndaya@cartafrica.org

Corresponding Author*: Wanangwa Chimwaza Manda, Kamuzu University of Health Sciences, Private Bag 360, Chichiri, Blantyre 3. Telephone: 265999445465. Email:wmanda@cartafrica.org

For peer review only

Abstract

Introduction

Targeting very young adolescents (VYAs) with sexual health (SH) interventions is increasingly being recognized as one of the strategies for addressing SH challenges in late adolescence. However, there is a dearth of literature regarding SH interventions implemented specifically for VYAs in sub-Saharan Africa (SSA). This scoping review aims to provide a summary of documented evidence on SH interventions that include VYAs in SSA, identify gaps in existing interventions, and provide recommendations for further programmatic work on SH for VYAs.

Methods and analysis

The methods for this scoping review will be guided by the framework proposed by Arksey and O'Malley and further enhanced by Levac et al. and the Joanna Briggs Institute. We will search electronic databases: Popline, EMBASE, PubMed, CINAHL, Dimensions, African Journals Online (AJOL), and specific summon-country-specific search. We will include published studies from SSA and only adolescent SH interventions published from the year 2003-2022. Furthermore, we will include programmatic and intervention literature that has not been published in peer-reviewed articles. The data will be charted using the Preferred Reporting Items for Systematic Review and Meta-Analysis Extension for Scoping Review (PRISMA-ScR). The data will then be collated and summarized.

Ethics and dissemination

The scoping review methodology involves putting together information from articles or grey literature that is either publicly available or shared by the authors, this study does not require ethical approval. Findings of this scoping review will be published in a scientific journal and presented at relevant scientific fora and conferences. This scoping review will provide a comprehensive overview of the evidence base of adolescent SH interventions for VYAs in SSA and will highlight critical gaps in the existing interventions and areas where further programmatic work is needed for VYAs in SSA.

Registration: https://archive.org/details/osf-registrations-gn538-v1

For peer review only - http://bmjopen.bmj.com/site/about/guidelines.xhtml

Keywords: Adolescent, very young adolescents, sexual health, Interventions, sub-Saharan Africa

For peer review only

For peer review only - http://bmjopen.bmj.com/site/about/guidelines.xhtml

Article Summary

Strengths and limitation of the study

- The search strategy includes a range of electronic databases with peer-reviewed literature, including grey literature, Popline, EMBASE, PubMed, CINAHL, Dimensions, African Journals Online (AJOL), Google, Directory of Open Access Journals (DOAJ), and summon-country specific search.
- Challenges are expected in finding published articles on VYAs SH interventions since research on VYAs has been limited until recent years.
- Since we are focusing on interventions and programs of which program implementers are usually not researchers, it is highly likely that some SH interventions or programs involving VYAs may not have been published in peer-reviewed articles.
- Stakeholders will be consulted to provide insights on interventions or programs that are not published or may have been missed by the scoping review.

Background

The United Nations Children's Fund (UNICEF) defines adolescence as the period between the ages of 10 and 19 (1). Adolescents are assigned into two categories of older adolescents (ages 15 to 19) and very young adolescents (VYAs) (1). Globally, there are 1.2 billion adolescents, and half are between the ages of 10 and 14 (2). Low and middle-income countries (LMICs) inhabit about 90% of these adolescents(3). During the period of adolescence, an individual undergoes rapid physical, biological, and intellectual changes as he or she transitions from childhood to adulthood (4,5). The period of adolescence is also regarded as the time in which the person advances from the onset of secondary sex features to that of sexual matureness e.g. increased facial, chest or pubic hair, and upper body muscle for boys, while girls show rounded figure/hips, breasts budding, and the onset of the menstrual cycle (6). Furthermore, mental functions and self-identity patterns transition from those of a young child to those of an autonomous adult (7). As the biological, cognitive, psychosocial, and emotional changes are happening the adolescents also experience changes in comprehending life, their relationships, spaces and supports they interact with across adolescence (6) In addition, it is during this period that new gender roles and sexual identities are established, various information is acquired, new attitudes are developed, and new behaviors and

For peer review only - http://bmjopen.bmj.com/site/about/guidelines.xhtml

relationships are tried (8,9). These developments and experiences put adolescents at risk of various SH challenges including infections that are transmitted sexually such as HIV, and early unintended pregnancies (10–16).

The majority of VYA are not yet engaging in penetrative sex, however, their sexual curiosities have begun and some are reported to be involved in non-coital activities such as kissing, cuddling, foreplay, and heavy petting (2,17–19). In addition, few studies conducted in SSA suggest that some VYA boys and girls engage in unsafe heterosexual vaginal sex either willingly or forced (20–23). Evidence from studies conducted in Malawi, Swaziland, Zimbabwe, and Kenya indicates that many VYA boys and girls were coerced into sex the first time they had sexual intercourse (24). These findings indicate that VYAs face various challenges related to their sexual health.

Most of the SH challenges occurring in early adolescence emanate from sexual risk behaviors that are linked to social-cultural factors including gender norms and socialization, socio-economic factors, and structural factors (8,11–16,24–26). For instance, expectations for gender roles that young boys should experiment sexually to prove their manhood promote their perceived superiority over girls, sexual aggressive behavior, and sexual risk-taking (24,27). On the other hand, the social expectation that girls should be passive, disadvantage them by reducing their power and agency to reject unwanted sex or negotiate safe sex (24,27). Recent studies in gender and education conducted in South Africa showcase how heterosexuality practices in primary schools promote oppressive behaviors against girls including violence, sexual coercive and humiliation (28,29). Furthermore, barriers to education such as school fees; long distances to school; cultural norms that promote boy's education only; and unfavorable school environments where girls encounter violence, may cause young girls to drop out of school, thereby increasing their likelihood of engaging risky sexual behaviors such as premarital sex and transactional sex (24,27). In addition, poverty which results in economic difficulties has been shown to be a cause of child marriage, adolescent pregnancy, unsafe sex, sexual activity involving multiple concurrent partners, and fewer educational possibilities (11–15,24,27). These risky sexual practices have been shown to have detrimental effects on the sexual and reproductive health of VYA girls, leading to adolescent pregnancies, unsafe abortions, and infections transmitted through sex such as HIV.

For peer review only - http://bmjopen.bmj.com/site/about/guidelines.xhtml

Every year about 3.9 million girls aged between 15 and 19 years have unsafe abortions(30). Around 2.1 million adolescents worldwide, including 770,000 VYA and 1.3 million older teenagers, were projected to have HIV in 2016(31). In LMICs, it is projected that 2 million VYA girls and 21 million girls within the age range of 15 and 19 give birth each year (32). Furthermore, In LMICs where most of the SH challenges among VYAs happen, the furtherance of adolescent sexual and reproductive health through putting more effort into positive youth development is increasing (18,27,33,34). As such, adolescent sexual and reproductive health programs are being implemented in various countries around the world through a variety of channels, such as peer education programs, youth centers, youth-friendly health services, and family life education (9,27,35). The majority of adolescent SH programs, however, neglect the SH needs of VYAs and instead concentrate on older adolescents (2,18,27,36). Not only are VYAs overlooked in SH interventions, but there is also a dearth of literature regarding sexual and reproductive health programs that include VYAs in SSA (SSA)(35,37). This scoping review aims to give a summary of documented evidence on SH interventions that include VYAs in SSA, identify gaps in existing interventions, and provide recommendations for further programmatic work on SH for VYAs in SSA. Since targeting VYAs with SH interventions is increasingly being recognized as one of the solutions to tackling SH challenges in late adolescence (8,27,38), it is envisaged that findings from this scoping review will contribute to the evidence that is required to develop evidence-based, culturally, and age-appropriate primary prevention programs and policies for addressing SH needs of VYA in SSA.

For peer review only

Specific objectives

1. To describe the types of SH interventions that have been implemented for VYAs in SSA
2. To describe the settings in which the SH interventions for VYAs have been implemented in SSA
3. To document the barriers and facilitators of implementing SH interventions for VYAs in SSA
4. To identify gaps in SH interventions implemented for VYAs in SSA
5. To provide recommendations for further programmatic work on SH for VYAs in SSA

Methods and Analysis

For peer review only - http://bmjopen.bmj.com/site/about/guidelines.xhtml

The framework suggested by Arksey and O'Malley, which has been improved by Levac et al. and the Joanna Briggs Institute, will serve as the basis for this scoping review's methodologies. (39,40). The stages in the framework include:

- Stage 1. Identifying the research question.
- Stage 2. Identifying relevant studies.
- Stage 3. Study selection.
- Stage 4. Charting the data.
- Stage 5. Collating, summarising, and reporting the result.
- Stage 6. Consultative forum.

Stage 1: Identifying the research questions

This scoping review has the following research questions:

Main question

What evidence is there for SH interventions that have been implemented for VYAs in SSA?

Specific questions

1. What types of SH interventions have been implemented for VYAs in SSA?
2. In what settings have these interventions been implemented?
3. What have been the implementation barriers and facilitators of these SH interventions for VYAs.
4. What are the gaps identified in these interventions?
5. What future programmatic work can be recommended on SH for very young adolescents in SSA?

Stage 2: Identifying relevant studies

To identify relevant studies for this review, a search for relevant articles and publications on adolescent sexual and reproductive health interventions in SSA will be conducted using several electronic databases including the Popline, EMBASE, PubMed, CINAHL, Dimensions, African Journals Online (AJOL) and Summon-country specific search. Additional searches of key terms

For peer review only - http://bmjopen.bmj.com/site/about/guidelines.xhtml

in Google Scholar and Directory of Open Access Journals (DOAJ) will be performed to find additional literature.

Several search techniques will be employed including phrase searching, Boolean operators (AND, OR, NOT), Truncation, Wildcats, Field search, Proximity search, citation chaining and filters to limit searches to inclusion criteria presented in this protocol. The final search strings will be presented in the report (refer to supplemental file 1). The search terms will include a combination of keywords and alternative terms such as: “Adolescent” OR “Very young Adolescents” OR “VYAs” OR “early adolescents” OR “Teenagers” OR “boys” OR “girls”, AND “Sexual and Reproductive health” OR “Reproductive health” OR “sexual health” OR “reproductive health care” OR “contraception” OR teen pregnancy” OR “sex education”, AND “Interventions” OR “Programming”, AND “Sub-Saharan Africa” OR “Country name of a Sub-Saharan country”. Using table 1 below, all researchers involved in the review will keep an up-to-date log of the number of articles found and the date found during each session of the literature search:

Table 1:Electronic search record

Date	Keyword searched	Search engine used	Number of publications retrieved

An academic librarian will be consulted to assist with identifying appropriate subject headings terms for the search and how to modify them for different databases that will be used. Relevant articles will be downloaded from the databases and uploaded into Mendeley (for importation and duplicates screening) first, then exported to Rayyan systematic review software for further screening of titles and abstracts.

3. Study Selection

According to the Arksey and O'Malley framework, the third stage of the process of the scoping review is to select studies to be included in the review (see figure 1 in the additional files section after the references). The first step in the third stage of the process will involve the screening of the titles and abstracts of the peer-reviewed articles and publications that were downloaded in the second stage. The articles and publications will be screened according to the criteria that were used

For peer review only - http://bmjopen.bmj.com/site/about/guidelines.xhtml

in the second stage which will include only studies from SSA; only adolescent sexual and reproductive health interventions and all interventions that include adolescents. The second step in this process will be a review of full-text articles and publications whose titles and abstracts have been deemed relevant for this review and the screening will be done by two reviewers. Should there be a disagreement between the two reviewers, the reviewers will consult with a third reviewer who will decide on whether to include the article/publication or not.

Inclusion criteria

The focus of the review will be peer reviewed articles and publications from the SSA from 2003-2022. Other criteria for inclusion will include language (only English articles and publications), type of Intervention (only sexual health and reproductive interventions) and study population (adolescents ages 10-19). These criteria were informed by the Population–Concept–Context (PCC) framework (see table 2) recommended by the Joanna Briggs Institute for scoping reviews to determine the eligibility of research and is recommended as less restrictive alternative to the PICO (Population, Intervention, Comparator and Outcome) mnemonic recommended for systematic reviews. Furthermore, we will include programmatic and intervention literature that has not been published in peer-reviewed articles.

Exclusion Criteria

The peer reviewed articles and publications from outside the SSA, publications not in English, including publications before 2003 and after 2022 will not be included in this review.

Table 2: Eligibility of the research question

PCC Element	Definition
Population	The population for this study will be all adolescents from ages 10 to 19.
Concept	All sexual health interventions targeting adolescents published between 2000 and 2022.
Context	Research articles and publications are limited to sub-Saharan Africa.

4. Charting the data

For peer review only - http://bmjopen.bmj.com/site/about/guidelines.xhtml

The fourth stage of the process of this scoping review will involve charting the data using Preferred Reporting Items for Systematic Review and Meta-Analysis Extension for Scoping review(PRISMA-ScR). This will involve the extraction of study characteristics and putting this information in a table or a chart. The study characteristics that will be extracted will include author, title, year of publication, and study location, type of intervention, duration of intervention, study population, and key findings (see table 3). The chart developed will be piloted by two reviewers separately to make sure that the chart is capturing information accurately. The whole process of extracting data from the studies will be done independently by two reviewers. To guarantee consistency between the reviewers, the charted data from the two will then be compared, and any inconsistencies will be further explored to reach a consensus.

Table 3: Sample data extraction table

1. Authors
2. Title
3. Year of publication
4. Country of Origin
5. Aims of the study
6. Sample size
7. Methodology
8. Intervention
9. Outcomes measured
10. Duration of the study
11. Key findings which match the review questions

5. Collating, summarizing, and reporting the result

This stage will involve collating, summarizing, and reporting the results from the process in Stage 4. This will provide summary information on what types of adolescent SRH interventions have been implemented for VYAs in SSA, the settings in which they were implemented, their effectiveness, and implementation challenges and facilitators. In addition, this process will also show SH interventions that have been under-researched and may require further investigation.

For peer review only - http://bmjopen.bmj.com/site/about/guidelines.xhtml

Stage 6: Consultation exercise

At this stage, we will involve relevant stakeholders working in the field of Adolescent SH in Malawi. The stakeholders will be engaged to provide valuable insight beyond our scoping review report. Since these stakeholders will be experts working in the field of adolescent SH we envisage that that they will add value to our work through providing additional references of SH interventions that the scoping review may have missed, suggesting existing SH interventions that have not been published, and giving insights to our findings. We will organize an in-person workshop in Malawi where we will present our findings to stakeholders and allow them to contribute their insights to our work.

Ethics and Dissemination

Since the scoping review methodology involves putting together information from articles that are publicly available, this study does not require ethical approval. To disseminate our findings, we will submit a manuscript for publication in a scientific journal. Furthermore, the findings of this scoping review will be presented in various scientific fora and conferences. It is anticipated that the scoping review will provide a comprehensive overview of the evidence base of adolescent SH interventions for VYAs and will highlight areas where evidence is inconclusive or missing. The review will also provide key information to policymakers and health professionals interested in planning, funding, and delivering evidence-based and effective SH for VYAs.

Patient and Public Involvement

No patient will be involved

Discussion

The proposed scoping review will generate a synthesis of SH programs or interventions implemented that included VYAs in SSA. For a long time, SH programs have focused on older adolescents aged 15 to 19 years overlooking VYAs (24,27). As such, literature is replete with programs or interventions for older adolescents (8). As efforts are being put into positive youth development to promote adolescent SH (27), attention is shifting to VYAs resulting in an increase in both VYAs SH research and programming over the past five years, globally (2,20,23,36,41,42).

For peer review only

For peer review only - http://bmjopen.bmj.com/site/about/guidelines.xhtml

However, there are very few SH interventions implemented among VYAs that have been documented in SSA (2,27).

Findings from this scoping review will be relevant for both policymakers and SH intervention programmers for VYAs in SSA. For instance, the synthesis of SH interventions that will be documented will showcase what SH interventions have been implemented for VYAs so far in SSA including the areas of focus, what works and what does not work in implementing these interventions. This knowledge is critical for developing evidence-based context-specific and culturally appropriate interventions for VYAs.

Furthermore, findings from this scoping review will be important for researchers working in the area of VYA SH as this scoping review may generate questions for further research around why some interventions work well and while others do not. In addition, is scoping review will contribute to knowledge by identifying gaps in SH interventions implemented for VYAs in SSA.

Definition of key terms

Adolescence: The period between the ages 10 and 19.

Adolescents: Young people from aged 10 to 19 years.

Very young adolescents: Adolescents aged 10 to 14 years.

Older adolescents: Adolescents aged 15 to 19 years.

Sexual health: a state of physical, emotional, mental, and social well-being in relation to sexuality; it is not merely the absence of disease, dysfunction, or infirmity.

Authors Contributions

For peer review only - http://bmjopen.bmj.com/site/about/guidelines.xhtml

WCM conceived of the idea, developed the research questions and study methods and contributed meaningfully to the drafting and editing. BK, AS, and SJ helped in developing the research questions and study methods, contributed meaningfully to the drafting and editing. YS and MK supervised the preparation of the protocol and critically reviewed the manuscript for important intellectual content. All authors approved the final manuscript.

Funding

This research (or "[initials of fellow]") will be supported by the Consortium for Advanced Research Training in Africa (CARTA). CARTA is jointly led by the African Population and Health Research Center and the University of the Witwatersrand and funded by the Carnegie Corporation of New York (Grant No. G-19-57145), Sida (Grant No:**54100113**), Uppsala Monitoring Center, Norwegian Agency for Development Cooperation (Norad), and by the Wellcome Trust [reference no. 107768/Z/15/Z] and the UK Foreign, Commonwealth & Development Office, with support from the Developing Excellence in Leadership, Training and Science in Africa (DELTAS Africa) programme. The statements made and views expressed are solely the responsibility of the Fellow. For the purpose of open access, the author has applied a CC BY public copyright licence to any Author Accepted Manuscript version arising from this submission.

Competing Interests

The authors declare that there are no competing interests

Availability of data and materials

All data will be published in a data repository once collected and can be accessed from the repository.

References

For peer review only - http://bmjopen.bmj.com/site/about/guidelines.xhtml

1. Unicef. Adolescence An Age of Opportunity [Internet]. Unicef. 2011. 137 p. Available from: www.unicef.org

2. Woog V, Kågesten A. The Sexual and Reproductive Health Needs of Very Young Adolescents Aged 10–14 in Developing Countries: What Does the Evidence Show? 2017;(May). Available from: https://www.guttmacher.org/sites/default/files/report_pdf/srh-needs-very-young-adolescents-report_0.pdf

3. Nagata JM, Ferguson BJ, Ross DA. Research Priorities for Eight Areas of Adolescent Health in Low- and Middle-Income Countries. J Adolesc Heal. 2016;59(1).

4. Dixon-Mueller R. How young is "too young"? Comparative perspectives on adolescent sexual, marital, and reproductive transitions. In: Studies in Family Planning. 2008. p. 247–62.

5. Morris JL, Rushwan H. Adolescent sexual and reproductive health: The global challenges. Int J Gynecol Obstet [Internet]. 2015;131:S40–52. Available from: http://dx.doi.org/10.1016/j.ijgo.2015.02.006

6. Kabiru CW, Beguy D, Undie C-C, Zulu EM, Ezeh AC. Transition into first sex among adolescents in slum and non-slum communities in Nairobi, Kenya. J Youth Stud [Internet]. 2010;13(4):453–71. Available from: http://www.tandfonline.com/doi/full/10.1080/13676261003801754

7. Rosen JE. Adolescent Health and Development (AHD) : A Resource Guide for World Bank Operations Staff and Government Counterparts. [Internet]. Washington DC; 2004. Available from: http://hdl.handle.net/10986/13635

8. Maina BW, Izugbara C, Sikweyiya Y, Nandongwa I, Kabiru CW. Gender Norms and Sexual Behavior among Very Young Adolescents in Sub-Saharan Africa. Routledge Handb African Demogr. 2021;330–48.

9. USAID, Institute for Reproductive Health. Reaching Very Young Adolescents (VYAs): Advancing Program, Research and Evaluation Practices. 2010; Available from:

For peer review only - http://bmjopen.bmj.com/site/about/guidelines.xhtml

For peer review only

http://irh.org/wp-content/uploads/2013/04/IRH_VYA_Technical_Consultation_2010_Meeting_Report_FINAL.pdf

10. Save the Children. Building Consensus to Address Very Young Adolescents ' Sexual and Reproductive Health : A Guide to Planning and Hosting a Technical Consultation [Internet]. 2015. Available from: https://toolkits.knowledgesuccess.org/sites/default/files/guide_for_developing_a_vya_consultation_june_2015.pdf

11. Daliana N, Farid N, Rus SC, Dahlui M, Al-sadat N, Aziz NA. Predictors of sexual risk behaviour among adolescents from welfare institutions in Malaysia : a cross sectional study. BMC Public Health [Internet]. 2014;14(Suppl 3):S9. Available from: http://www.biomedcentral.com/1471-2458/14/S3/S9

12. Albert, B., Brown, S., & Flanigan C. 14 and Younger: The Sexual Behavior of Young Adolescents (Summary) [Internet]. Washington, DC: National Campaign to Prevent Teen Pregnancy; 2003. Available from: https://files.eric.ed.gov/fulltext/ED477795.pdf

13. Tangmunkongvorakul A, Carmichael G, Banwell C, Utomo ID, Sleigh A. Sexual perceptions and practices of young people in Northern Thailand. J Youth Stud [Internet]. 2011;14(3):315. Available from: http://www.ncbi.nlm.nih.gov/pubmed/22319025

14. Upadhyay UD, Hindin MJ. Do Perceptions of Friends' Behaviors Affect Age at First Sex? Evidence from Cebu, Philippines. J Adolesc Heal. 2006;39(4):570–7.

15. Clark S. Early Marriage and HIV Risks in Sub-Saharan Africa. Stud Fam Plann [Internet]. 2004;35(3):149–60. Available from: http://doi.wiley.com/10.1111/j.1728-4465.2004.00019.x

16. Mehta SD, Seeley J. Grand Challenges in Adolescent Sexual and Reproductive Health. Front Reprod Heal. 2020;2(June):2–5.

17. Dixon-Mueller R. Starting young: Sexual initiation and HIV prevention in early adolescence. Vol. 13, AIDS and Behavior. 2009. p. 100–9.

For peer review only

For peer review only - http://bmjopen.bmj.com/site/about/guidelines.xhtml

18. Brady M. Calling attention to young adolescents: Building the evidence base to inform policies and programs [Internet]. Transitions to Adulthood. 2011. Available from: https://www.popcouncil.org/uploads/pdfs/TABriefs/37_VeryYoungAdolescents.pdf

19. Maina BW, Orindi BO, Sikweyiya Y, Kabiru CW. Gender norms about romantic relationships and sexual experiences among very young male adolescents in Korogocho slum in Kenya. Int J Public Health [Internet]. 2020;65(4):497–506. Available from: https://doi.org/10.1007/s00038-020-01364-9

20. Bankole A, Biddlecom A, Guiella G, Singh S, Zulu E. Sexual behavior, knowledge and information sources of very young adolescents in four sub-Saharan African countries. Afr J Reprod Health [Internet]. 2007;11(3):28–43. Available from: http://www.ncbi.nlm.nih.gov/pubmed/18458739%5Cnhttp://www.pubmedcentral.nih.gov/articlerender.fcgi?artid=PMC2367131

21. Atuyambe LM, Kibira SPS, Bukenya J, Muhumuza C, Apolot RR, Mulogo E. Understanding sexual and reproductive health needs of adolescents: evidence from a formative evaluation in Wakiso district, Uganda. Reprod Health [Internet]. 2015;12:35. Available from: http://www.ncbi.nlm.nih.gov/pubmed/25896066%5Cnhttp://www.pubmedcentral.nih.gov/articlerender.fcgi?artid=PMC4416389

22. Seifu A, Fantahun M, Worku A. Reproductive health needs of out-of-school adolescents : A cross-sectional comparative study of rural and urban areas in northwest Ethiopia. EthiopJHealth Dev. 2006;20(1):17.

23. Ortiz-Echevarria L, Greeley M, Bawoke T, Zimmerman L, Robinson C, Schlecht J. Understanding the unique experiences, perspectives and sexual and reproductive health needs of very young adolescents: Somali refugees in Ethiopia. Confl Health. 2017;11(Suppl 1).

24. Woog, V. and Kagesten A. The Sexual and Reproductive Health Needs of Very Young Adolescents In Developing Countries. Guttmarcher Inst. 2017;(May).

25. Igras S, Yahner M, Ralaison H, Rakotovao JP, Favero R, Andriantsimietry S, et al.

For peer review only - http://bmjopen.bmj.com/site/about/guidelines.xhtml

Reaching the youngest moms and dads: A socio-ecological view of actors and factors influencing first-time young parents' use of sexual and reproductive health services in Madagascar. Afr J Reprod Health [Internet]. 2019;23(3):19–29. Available from: https://pesquisa.bvsalud.org/gim/resource/en/biblio-1258537

26. AOU O, JU O. Sexual Activity and Contraceptive Use among Female Adolescents-A Report from Port Harcount, Nigeria. Africa J Reprod Heal. 2000;4(1):40–7.

27. Igras SM, Macieira M, Murphy E, Lundgren R. Investing in very young adolescents' sexual and reproductive health. Glob Public Health [Internet]. 2014;9(5):555–69. Available from: http://www.ncbi.nlm.nih.gov/pubmed/24824757%5Cnhttp://www.pubmedcentral.nih.gov/articlerender.fcgi?artid=PMC4066908

28. Morison T, Macleod CI, Lynch I. 'My friends would laugh at me': embedding the dominant heterosexual script in the talk of primary school students. Gend Educ [Internet]. 2022;34(3):329–45. Available from: https://doi.org/10.1080/09540253.2021.1929856

29. Govender N, Bhana D. Girls and the negotiation of heterosexual femininities in the primary school. In: Palgrave Studies in Gender and Education [Internet]. 2021. p. 113–33. Available from: https://link.springer.com/10.1007/978-3-030-69988-8_5

30. Franjić S. Adolescent Pregnancy is a Serious Social Problem. J Gynecol Res Obstet. 2018;4(1):5–8.

31. UNFPA. Adolescent sexual and reproductive health. United Nations Popul Fund [Internet]. 2014;(August):1–18. Available from: http://www.unfpa.org/resources/adolescent-sexual-and-reproductive-health

32. Darroch JE, Woog V, Bankole A. ADDING IT UP : Costs and Benefits of Meeting the Contraceptive Needs of Adolescents. New York Guttmacher Inst. 2016;(May):1–16.

33. Blum et al. A conceptual framework for early adolescence: a platform for research. Int J Adolesc Med Heal. 2015;33(4):395–401.

34. Sommer M. An overlooked priority: Puberty in sub-Saharan Africa. Am J Public Health.

For peer review only - http://bmjopen.bmj.com/site/about/guidelines.xhtml

2011;101(6):979–81.

35. World Health Organization. The sexual and reproductive health of young adolescents in developing countries:Reviewing the evidence, identifying research gaps, and moving the agenda [Internet]. WHO. 2010. Available from: http://apps.who.int/iris/bitstream/handle/10665/70569/WHO_RHR_11.11_eng.pdf;jsessionid=8BF57F4C2F2CF2897A8D118A83BA8324?sequence=1

36. McCarthy K, Brady M, Hallman K. Investing When It Counts: Reviewing the Evidence and Charting a Course of Research and Action for Very Young Adolescents. 2016;1–69.

37. Lee C, Aules Y, Sami S, Lar PK, Schlect J, Robinson C. Sexual and reproductive health needs and risks of very young adolescent refugees and migrants from Myanmar living in Thailand. Confl Health. 2017;11(Suppl 1).

38. Odimegwu CO, Adewoyin Y. The Routledge Handbook of African Demography [Internet]. Katz WF, Assmann PF, editors. The Routledge Handbook of Demography. London: Routledge; 2021. 1–648 p. Available from: https://www.taylorfrancis.com/books/9780429508561

39. Arksey H, O'Malley L. Scoping studies: Towards a methodological framework. Int J Soc Res Methodol Theory Pract. 2005;8(1):19–32.

40. Fallis A. Reviewer's Manual. J Chem Inf Model [Internet]. 2013;53(9):1689–99. Available from: www.joannabriggs.org

41. Mmari K, A M, Blum RW, Ph D, H MP, Atnafou R, et al. Exploration of Gender Norms and Socialization Among Early Adolescents : The Use of Qualitative Methods for the Global Early Adolescent Study. J Adolesc Heal [Internet]. 2017;61(4):S12–8. Available from: http://dx.doi.org/10.1016/j.jadohealth.2017.07.006

42. Essop R, Tolla T, Lynch I, Makoae M. 'They tell you about the risks': Exploring sources of sexuality education among very young adolescents in rural Mpumalanga. SAJCH South African J Child Heal. 2018;12(Special Issue):S36–9.

For peer review only

For peer review only - http://bmjopen.bmj.com/site/about/guidelines.xhtml

Figure Legend

Figure 1 is a flow diagram describing the process of articles and publications being reviewed and selected. 1. ***Identification***: articles will be identified through a database search and through searching of other sources e.g. grey literature; 2***. Screening***: the identified records will be screened and duplicates removed; 3. ***Eligibility***: Full-text articles will be screened for eligibility and those not falling within the inclusion criteria will be excluded; 4. ***Included***: studies that will finally be included for the review.

For peer review only

For peer review only - http://bmjopen.bmj.com/site/about/guidelines.xhtml

Figure 1: Study selection process

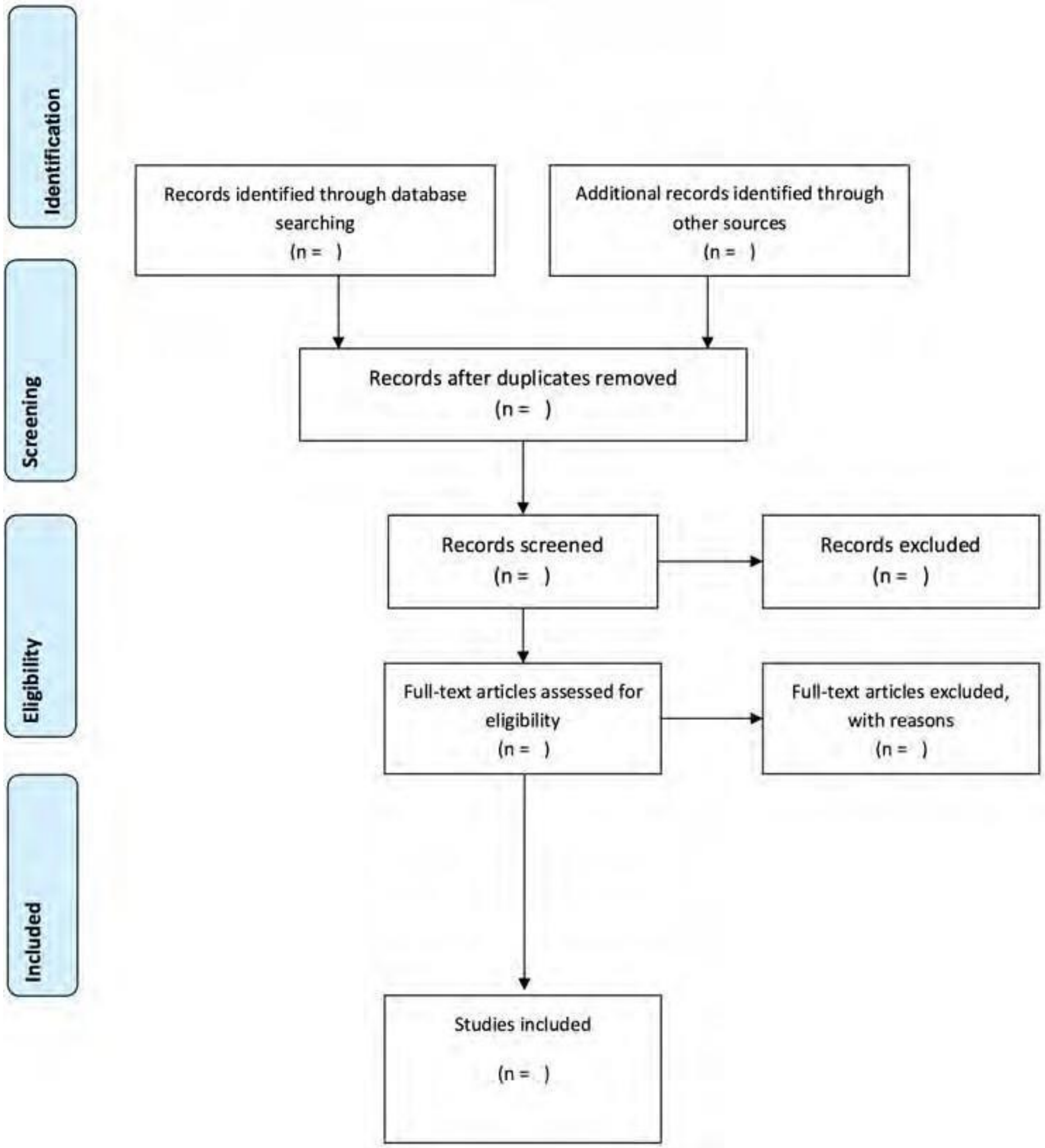

Figure 1. Flow diagram describing process of articles and publications being reviewed and selected

Below is summary of initial searches done

**Date**	**Search engine used**	**Number of publications retrieved**
17/10/21	CINAHL(Ebsco)	1,927
17/09/21	Cochrane Library	1,007
17/09/21	Ovid MEDLINE	2,055
17/09/21	Ovid EMBASE	2,576
17/10/21	Global Index Medicus	1,752

Embase Classic+Embase <1947 to 2021 October 11>

1	exp adolescent behavior/ or exp adolescent mother/ or exp adolescent development/ or exp adolescent health/ or exp adolescent parent/ or exp adolescent sexual behavior/ or exp adolescent disease/ or exp adolescent pregnancy/ or exp adolescent/ or exp adolescent father/	1794522
2	exp sexual health/ or exp sexual behavior/ or exp sexuality/ or exp sexually transmitted disease/ or exp family planning/ or exp sexual education/	429495
3	exp pregnancy/ or exp reproductive health/	856202
4	exp child health care/	100563
5	exp Human immunodeficiency virus infection/	392472
6	exp adolescent pregnancy/	9963
7	(''sexual* health'' or sex* education or ''reproduct* health'' or pregnan* or contracept* or ''familly planning'' or HIV infections'' or ''Adolescen* behaviors'' or ''sexual behavior'' or ''child health care'' or ''adolescent development'' or ''adolescent behavior'' or ''adolescent mother'' or ''adolescent development'' or ''adolescent health'' or ''adolescent parent'' or ''adolescent disease'' or ''adolescent father'').tw,ti,ab.	887005
8	1 or 2 or 3 or 4 or 5 or 6 or 7	3588786

For peer review only

9	exp program development/ or exp program evaluation/	51829
10	exp health promotion/ or exp health education/	351668
11	(comprehensive or integrated or multilevel or multifaceted or multi component or multifaceted or multicomponent or multidimensional or multidimensional or holistic or ''holistic health'' or ''Community Networks'' or ''youth development program*'' or ''youth program*'' or ''teen program*'' or adolescent program* or ''opportunity programme'' or ''health promot*'' or ''program evaluation'').tw,ti,ab.	988657
12	9 or 10 or 11	1325483
13	8 and 12	172938
14	(teen* or youth* or adolescen* or juvenile* or (young adj2 (adult* or person* or individual* or people* or population* or man or men or wom*n)) or youngster* or first-grader* or second-grader* or third-grader* or fourth-grader* or fifth-grader* or sixth-grader* or seventh-grader* or highschool* or college* or ((secondary or high*) adj2 (school* or education))).tw,ti,ab.	1126352
15	13 and 14	49877
16	exp Africa, Central/ or exp Africa, Eastern/ or exp Africa, Western/ or exp Africa/ or exp "Africa South of the Sahara"/ or exp Africa, Southern/ or exp South Africa/	387913
17	15 and 16	2789
18	limit 17 to (human and yr="2000 -Current")	2576

Ovid MEDLINE: Epub Ahead of Print, In-Process & Other Non-Indexed Citations, Ovid MEDLINE® Daily and Ovid MEDLINE® <1946-Present>

1	exp Adolescent Behavior/ or exp Adolescent Health Services/ or exp Adolescent/ or exp Adolescent Health/ or exp Adolescent Development/	2127695
2	exp Sexual Health/ or exp Reproductive Health/ or exp Sexual Behavior/ or exp Sex Education/	122155
3	exp Reproductive Health/ or exp "Delivery of Health Care"/ or exp Family Planning Services/	1169678
4	exp Sexual Behavior/	113345

For peer review only

5	exp Pregnancy in Adolescence/	8244
6	exp HIV Infections/	296978
7	(''sexual* health'' or sex* education or ''reproduct* health'' or pregnan* or contracept* or ''familly planning'' or HIV infections'' or ''Adolescen* behaviors'' or ''sexual behavior'' or ''child health care'' or ''adolescent development'' or ''adolescent behavior'' or ''adolescent mother'' or ''adolescent development'' or ''adolescent health'' or ''adolescent parent'' or ''adolescent disease'' or ''adolescent father'').tw,ti,ab.	637818
8	1 or 2 or 3 or 4 or 5 or 6 or 7	3919310
9	exp program development/ or exp program evaluation/	100752
10	exp health promotion/ or exp health education/	254114
11	(comprehensive or integrated or multilevel or multifaceted or multi component or multifaceted or multicomponent or multidimensional or multidimensional or holistic or ''holistic health'' or ''Community Networks'' or ''youth development program*'' or ''youth program*'' or ''teen program*'' or adolescent program* or ''opportunity programme'' or ''health promot*'' or ''program evaluation'').tw,ti,ab.	779773
12	9 or 10 or 11	1072828
13	8 and 12	282250
14	(teen* or youth* or adolescen* or juvenile* or (young adj2 (adult* or person* or individual* or people* or population* or man or men or wom*n)) or youngster* or first-grader* or second-grader* or third-grader* or fourth-grader* or fifth-grader* or sixth-grader* or seventh-grader* or highschool* or college* or ((secondary or high*) adj2 (school* or education))).tw,ti,ab.	783907
15	13 and 14	48396
16	exp Africa, Central/ or exp Africa, Eastern/ or exp Africa, Western/ or exp Africa/ or exp "Africa South of the Sahara"/ or exp Africa, Southern/ or exp South Africa/	290672
17	15 and 16	2447
18	limit 17 to (humans and yr="2000 -Current")	2055

For peer review only

Cochrane

ID	Search	Hits
#1	MeSH descriptor: [Adolescent] explode all trees	107314
#2	MeSH descriptor: [Pregnancy in Adolescence] explode all trees	199
#3	MeSH descriptor: [Adolescent Behavior] explode all trees	1504
#4	MeSH descriptor: [Sexual Health] explode all trees	69
#5	MeSH descriptor: [Family Planning Services] explode all trees	256
#6	MeSH descriptor: [Reproductive Health] explode all trees	91
#7	MeSH descriptor: [HIV Infections] explode all trees	13092
#8	(''sexual* health'' or sex* education or ''reproduct* health'' or pregnan* or contracept* or ''familly planning'' or HIV infections'' or ''Adolescen* behaviors'' or ''sexual behavior'' or ''child health care'' or ''adolescent development'' or ''adolescent behavior'' or ''adolescent mother'' or ''adolescent development'' or ''adolescent health'' or ''adolescent parent'' or ''adolescent disease'' or ''adolescent father''):ti,ab,kw	164702
#9	#1 OR #2 OR #3 OR #4 OR #5 OR #6 OR #7 OR #8	226282
#10	MeSH descriptor: [Program Development] explode all trees	730
#11	MeSH descriptor: [Program Evaluation] explode all trees	6425
#12	(comprehensive or integrated or multilevel or multifaceted or multi component or multifaceted or multicomponent or multidimensional or multidimensional or holistic or ''holistic health'' or ''Community Networks'' or ''youth development program*'' or ''youth program*'' or ''teen program*'' or adolescent program* or ''opportunity programme'' or ''health promot*'' or ''program evaluation''):ti,ab,kw	97084
#13	#10 OR #11 OR #12	97312
#14	#9 AND #13	27099
#15	((teen* or youth* or adolescen* or juvenile* or (young adj2 (adult* or person* or individual* or people* or population* or man or men or wom*n)) or youngster* or first-grader* or second-grader* or third-grader* or fourth-grader* or fifth-grader* or sixth-grader* or seventh-grader* or highschool* or college* or ((secondary or high*) adj2 (school* or education)))):ti,ab,kw	166583

For peer review only

#16	#14 AND #15	18460
#17	(sub-Sahara* Africa OR West Africa OR East Africa OR Central Africa OR South* Africa OR SSA OR Cameroon OR "Central African Republic" OR Chad OR Congo OR "Democratic Republic of the Congo" OR Gabon OR Burundi OR Djibouti OR Eritrea OR Ethiopia OR Kenya OR Rwanda OR Somalia OR Sudan OR Tanzania OR Uganda OR Angola OR Botswana OR Lesotho OR Malawi OR Mozambique OR Namibia OR Swaziland OR Zambia OR Zimbabwe OR Benin OR Burkina Faso OR Cape Verde OR Cote d'Ivoire OR Gambia OR Ghana OR Guinea OR Equatorial Guinea OR Guinea-Bissau OR Liberia OR Mali OR Mauritania OR Niger OR Nigeria OR Senegal OR Sierra Leone OR Togo OR Comoros OR Madagascar OR Mauritius OR Seychelles OR Solomon Islands OR Marshall Islands OR Sao Tome and Principe):ti,ab,kw	20737
#18	#16 AND #17 with Publication Year from 2000 to 2021, with Cochrane Library publication date Between Jan 2000 and Dec 2021, in Trials (Word variations have been searched)	1007

Ebsco

Accessibility Information and Tips

Print Search History

MY EBSCOhost

Sunday, October 17, 2021 9:48:02 AM

#	Query	Limiters/Expanders	Last Run Via	Results
S23	S18 AND S19	Limiters - Published Date: 20000101-20211231 Expanders - Apply equivalent subjects Search modes - Boolean/Phrase	Interface - EBSCOhost Research Databases Search Screen - Advanced Search Database - CINAHL Plus with Full Text	1,927

For peer review only

S22	S18 AND S19	Expanders - Apply equivalent subjects<br>Search modes - Boolean/Phrase	Interface - EBSCOhost Research Databases<br>Search Screen - Advanced Search<br>Database - CINAHL Plus with Full Text	1,952
S21	S18 AND S19	Limiters - Published Date: 20000101-20211231<br>Expanders - Apply equivalent subjects<br>Search modes - Boolean/Phrase	Interface - EBSCOhost Research Databases<br>Search Screen - Advanced Search<br>Database - CINAHL Plus with Full Text	1,927
S20	S18 AND S19	Expanders - Apply equivalent subjects<br>Search modes - Boolean/Phrase	Interface - EBSCOhost Research Databases<br>Search Screen - Advanced Search<br>Database - CINAHL Plus with Full Text	1,952
S19	(MH "South Africa") OR (MH "Namibia") OR (MH "Africa South of the Sahara")	Expanders - Apply equivalent subjects<br>Search modes - Boolean/Phrase	Interface - EBSCOhost Research Databases<br>Search Screen - Advanced Search<br>Database - CINAHL Plus with Full Text	24,636
S18	S16 AND S17	Expanders - Apply equivalent subjects<br>Search modes - Boolean/Phrase	Interface - EBSCOhost Research Databases<br>Search Screen - Advanced Search<br>Database - CINAHL Plus with Full Text	137,234
S17	TX (teen* OR youth* OR adolescen* OR juvenile* OR (young N2 (adult* OR person* OR individual* OR people* OR population* OR man OR men OR wom*n)) OR youngster* OR first-grader* OR second-grader* OR third-grader* OR fourth-grader* OR	Expanders - Apply equivalent subjects<br>Search modes - Boolean/Phrase	Interface - EBSCOhost Research Databases<br>Search Screen - Advanced Search<br>Database - CINAHL Plus with Full Text	1,853,657

For peer review only

	fifth-grader* OR sixth-grader* OR seventh-grader* OR highschool* OR college* OR ((secondary or high*) N2 (school* OR education)))			
S16	S11 AND S15	Expanders - Apply equivalent subjects Search modes - Boolean/Phrase	Interface - EBSCOhost Research Databases Search Screen - Advanced Search Database - CINAHL Plus with Full Text	171,808
S15	S12 OR S13 OR S14	Expanders - Apply equivalent subjects Search modes - Boolean/Phrase	Interface - EBSCOhost Research Databases Search Screen - Advanced Search Database - CINAHL Plus with Full Text	746,708
S14	TX comprehensive OR integrated OR multilevel OR multifaceted OR ''multi component'' OR multifaceted OR multicomponent OR multidimensional OR multidimensional'' OR holistic OR ''holistic health'' OR ''Community Networks'' OR ''youth development program*'' OR ''youth program*'' OR ''teen program*'' OR ''adolescent program*'' OR ''opportunity programme'' OR ''health promot*'' OR ''program evaluation''	Expanders - Apply equivalent subjects Search modes - Boolean/Phrase	Interface - EBSCOhost Research Databases Search Screen - Advanced Search Database - CINAHL Plus with Full Text	731,345
S13	(MH "Program Evaluation")	Expanders - Apply equivalent subjects Search modes - Boolean/Phrase	Interface - EBSCOhost Research Databases Search Screen - Advanced Search	44,498

For peer review only

			Database - CINAHL Plus with Full Text	
S12	(MH "Program Development")	Expanders - Apply equivalent subjects Search modes - Boolean/Phrase	Interface - EBSCOhost Research Databases Search Screen - Advanced Search Database - CINAHL Plus with Full Text	27,514
S11	S1 OR S2 OR S3 OR S4 OR S5 OR S6 OR S7 OR S8 OR S9 OR S10	Expanders - Apply equivalent subjects Search modes - Boolean/Phrase	Interface - EBSCOhost Research Databases Search Screen - Advanced Search Database - CINAHL Plus with Full Text	1,051,780
S10	TX ''sexual* health'' OR ''sex* education'' OR ''reproduct* health'' OR pregnan* OR contracept* OR ''familly planning'' OR ''HIV infections'' OR ''Adolescen* behaviors'' OR ''sexual behavior'' OR ''child health care'' OR ''adolescent development'' OR ''adolescent behavior'' OR ''adolescent mother'' OR ''adolescent development'' OR ''adolescent health'' OR ''adolescent parent'' OR ''adolescent disease'' OR ''adolescent father''	Expanders - Apply equivalent subjects Search modes - Boolean/Phrase	Interface - EBSCOhost Research Databases Search Screen - Advanced Search Database - CINAHL Plus with Full Text	596,080
S9	(MH "HIV Infections+") OR (MH "Human Immunodeficiency Virus+")	Expanders - Apply equivalent subjects Search modes - Boolean/Phrase	Interface - EBSCOhost Research Databases Search Screen - Advanced Search Database - CINAHL Plus with Full Text	92,927
S8	(MH "Family Planning") OR (MH "Family	Expanders - Apply equivalent subjects	Interface - EBSCOhost Research Databases	7,446

For peer review only

	Planning, Natural") OR (MH "Family Planning: Unplanned Pregnancy (Iowa NIC)") OR (MH "Family Planning: Contraception (Iowa NIC)") OR (MH "Family Planning (Omaha)")	Search modes - Boolean/Phrase	Search Screen - Advanced Search Database - CINAHL Plus with Full Text	
S7	(MH "Sexual Behavior")	Expanders - Apply equivalent subjects Search modes - Boolean/Phrase	Interface - EBSCOhost Research Databases Search Screen - Advanced Search Database - CINAHL Plus with Full Text	694
S6	(MH "Reproductive Health")	Expanders - Apply equivalent subjects Search modes - Boolean/Phrase	Interface - EBSCOhost Research Databases Search Screen - Advanced Search Database - CINAHL Plus with Full Text	8,101
S5	(MH "Sex Education")	Expanders - Apply equivalent subjects Search modes - Boolean/Phrase	Interface - EBSCOhost Research Databases Search Screen - Advanced Search Database - CINAHL Plus with Full Text	5,793
S4	(MH "Sexual Health") OR (MH "Sexually Transmitted Diseases, Viral")	Expanders - Apply equivalent subjects Search modes - Boolean/Phrase	Interface - EBSCOhost Research Databases Search Screen - Advanced Search Database - CINAHL Plus with Full Text	7,940
S3	(MH "Sexually Transmitted Diseases, Viral")	Expanders - Apply equivalent subjects Search modes - Boolean/Phrase	Interface - EBSCOhost Research Databases Search Screen - Advanced Search Database - CINAHL Plus with Full Text	393
S2	(MH "Adolescent Parents") OR (MH	Expanders - Apply equivalent subjects	Interface - EBSCOhost Research Databases	27,742

For peer review only

	"Adolescent Mothers") OR (MH "Adolescent Health Services") OR (MH "Adolescent Fathers") OR (MH "Adolescent Behavior") OR (MH "Adolescent Health")	Search modes - Boolean/Phrase	Search Screen - Advanced Search Database - CINAHL Plus with Full Text	
S1	(MH "Adolescent Parents") OR (MH "Child Development: Adolescence (12-17 Years) (Iowa NOC)") OR (MH "Adolescence") OR (MH "Pregnancy in Adolescence") OR (MH "Maternal Age 14 and Under")	Expanders - Apply equivalent subjects Search modes - Boolean/Phrase	Interface - EBSCOhost Research Databases Search Screen - Advanced Search Database - CINAHL Plus with Full Text	563,120

Global Index Medicus

search		
1	tw:((tw:(adolescent)) OR (tw:("family planning")) OR (tw:("sexual health")) OR (tw:(adolescent pregnancy")) OR (tw:("adolescent behavior")) OR (tw:(sexuality)) OR (tw:("sexual behavior")) OR (tw:("hiv infection"))) AND (collection_gim:("AIM")) AND (year_cluster:[2000 TO 2021])	1752

For peer review only

Preferred Reporting Items for Systematic reviews and Meta-Analyses extension for Scoping Reviews (PRISMA-ScR) Checklist

SECTION	ITEM	PRISMA-ScR CHECKLIST ITEM	REPORTED ON PAGE #
**TITLE**			
Title	1	Identify the report as a scoping review.	1
**ABSTRACT**			
Structured summary	2	Provide a structured summary that includes (as applicable): background, objectives, eligibility criteria, sources of evidence, charting methods, results, and conclusions that relate to the review questions and objectives.	2
**INTRODUCTION**			
Rationale	3	Describe the rationale for the review in the context of what is already known. Explain why the review questions/objectives lend themselves to a scoping review approach.	4
Objectives	4	Provide an explicit statement of the questions and objectives being addressed with reference to their key elements (e.g., population or participants, concepts, and context) or other relevant key elements used to conceptualize the review questions and/or objectives.	5-6
**METHODS**			
Protocol and registration	5	Indicate whether a review protocol exists; state if and where it can be accessed (e.g., a Web address); and if available, provide registration information, including the registration number.	2
Eligibility criteria	6	Specify characteristics of the sources of evidence used as eligibility criteria (e.g., years considered, language, and publication status), and provide a rationale.	8
Information sources*	7	Describe all information sources in the search (e.g., databases with dates of coverage and contact with authors to identify additional sources), as well as the date the most recent search was executed.	6-7
Search	8	Present the full electronic search strategy for at least 1 database, including any limits used, such that it could be repeated.	7
Selection of sources of evidence†	9	State the process for selecting sources of evidence (i.e., screening and eligibility) included in the scoping review.	7-8
Data charting process‡	10	Describe the methods of charting data from the included sources of evidence (e.g., calibrated forms or forms that have been tested by the team before their use, and whether data charting was done independently or in duplicate) and any processes for obtaining and confirming data from investigators.	8-9
Data items	11	List and define all variables for which data were sought and any assumptions and simplifications made.	9
Critical appraisal of individual sources of evidence§	12	If done, provide a rationale for conducting a critical appraisal of included sources of evidence; describe the methods used and how this information was used in any data synthesis (if appropriate).	Not done yet

For peer review only - http://bmjopen.bmj.com/site/about/guidelines.xhtml

SECTION	ITEM	PRISMA-ScR CHECKLIST ITEM	REPORTED ON PAGE #
Synthesis of results	13	Describe the methods of handling and summarizing the data that were charted.	10
**RESULTS**			
Selection of sources of evidence	14	Give numbers of sources of evidence screened, assessed for eligibility, and included in the review, with reasons for exclusions at each stage, ideally using a flow diagram.	7, 18
Characteristics of sources of evidence	15	For each source of evidence, present characteristics for which data were charted and provide the citations.	Not yet done
Critical appraisal within sources of evidence	16	If done, present data on critical appraisal of included sources of evidence (see item 12).	Not yet done
Results of individual sources of evidence	17	For each included source of evidence, present the relevant data that were charted that relate to the review questions and objectives.	Not yet done
Synthesis of results	18	Summarize and/or present the charting results as they relate to the review questions and objectives.	Not yet done
**DISCUSSION**			
Summary of evidence	19	Summarize the main results (including an overview of concepts, themes, and types of evidence available), link to the review questions and objectives, and consider the relevance to key groups.	Not yet done
Limitations	20	Discuss the limitations of the scoping review process.	3
Conclusions	21	Provide a general interpretation of the results with respect to the review questions and objectives, as well as potential implications and/or next steps.	Not yet done
**FUNDING**			
Funding	22	Describe sources of funding for the included sources of evidence, as well as sources of funding for the scoping review. Describe the role of the funders of the scoping review.	16

JBI = Joanna Briggs Institute; PRISMA-ScR = Preferred Reporting Items for Systematic reviews and Meta-Analyses extension for Scoping Reviews.

* Where *sources of evidence* (see second footnote) are compiled from, such as bibliographic databases, social media platforms, and Web sites.

† A more inclusive/heterogeneous term used to account for the different types of evidence or data sources (e.g., quantitative and/or qualitative research, expert opinion, and policy documents) that may be eligible in a scoping review as opposed to only studies. This is not to be confused with *information sources* (see first footnote).

‡ The frameworks by Arksey and O'Malley (6) and Levac and colleagues (7) and the JBI guidance (4, 5) refer to the process of data extraction in a scoping review as data charting.

§ The process of systematically examining research evidence to assess its validity, results, and relevance before using it to inform a decision. This term is used for items 12 and 19 instead of "risk of bias" (which is more applicable to systematic reviews of interventions) to include and acknowledge the various sources of evidence that may be used in a scoping review (e.g., quantitative and/or qualitative research, expert opinion, and policy document).

From: Tricco AC, Lillie E, Zarin W, O'Brien KK, Colquhoun H, Levac D, et al. PRISMA Extension for Scoping Reviews (PRISMAScR): Checklist and Explanation. Ann Intern Med. 2018;169:467–473. doi: 10.7326/M18-0850.

For peer review only - http://bmjopen.bmj.com/site/about/guidelines.xhtml
